# Optimization and production of a GLP-grade recombinant Sm29 vaccine against schistosomiasis: From expression to preclinical assessment

**DOI:** 10.1371/journal.pntd.0014617

**Published:** 2026-08-07

**Authors:** Juvana M. Andrade, Fábio Mambelli, Dharliton S. Gomes, Monique F. S. Souza, Júlia S. Fahel, Rodrigo C. O. Sanches, Fábio V. Marinho, Natália Salazar, Isabela P. Gomes, Vívian T. Martins, Edgar M. Carvalho, Mariana T. Q. de Magalhaes, Santuza R. Teixeira, Sergio C. Oliveira

**Affiliations:** 1 Departamento de Bioquímica e Imunologia, Instituto de Ciências Biológicas, Universidade Federal de Minas Gerais, Belo Horizonte, Minas Gerais, Brazil; 2 Departamento de Imunologia, Instituto de Ciências Biomédicas, Universidade de São Paulo, São Paulo, Brazil; 3 Departamento de Clínica e Cirurgia Veterinárias, Escola de Veterinária, Universidade Federal de Minas Gerais, Belo Horizonte, Minas Gerais, Brazil; 4 Centro de Tecnologia de Vacinas, Universidade Federal de Minas Gerais, Parque Tecnológico de Belo Horizonte, Minas Gerais, Brazil; 5 Laboratório de Pesquisas Clínicas, Instituto Gonçalo Moniz, FIOCRUZ, Salvador, Brazil; 6 Instituto Nacional de Ciências e Tecnologia-Doenças Tropicais, Salvador, Brazil; 7 Laboratório de Biofísica de Macromoleculas, Programa de Pós-graduação em Quimica, Instituto Militar de Engenharia, IME, Rio de Janeiro, Rio de Janeiro, Brazil; George Washington University School of Medicine and Health Sciences, UNITED STATES OF AMERICA

## Abstract

Schistosomiasis remains a major neglected tropical disease, and the development of a safe, effective vaccine is a global priority. Sm29, a *Schistosoma mansoni* surface antigen associated with naturally acquired resistance, has emerged as a promising vaccine candidate; however, its translational advancement requires both high-quality antigen production and evaluation with clinically acceptable adjuvants. Here, we report a Good Laboratory Practice-aligned process for generating tag-free recombinant Sm29 in *Escherichia coli*, including multi-step anion-exchange chromatography followed by subsequent removal of residual impurities. Analytical validation demonstrated high purity, minimal host cell proteins and residual DNA and low endotoxin levels compliant with international regulatory standards. We then assessed the immunogenicity and protective efficacy of recombinant Sm29 formulated with alum or the squalene-based emulsion (CTVad1) in a murine model of *S. mansoni* infection. Both formulations elicited robust humoral immune responses, characterized by high titers of total IgG, IgG1, and IgG2c, and moderate levels of IgG3 and IgE anti-Sm29. Regarding cytokines, Sm29 formulated with alum induced a mixed Th1/Th2 immunological profile while Sm29 adjuvanted with CTVad1 engendered a Th2-like response. Following cercarial challenge, CTVad1 + Sm29 or Alum+Sm29 vaccinated mice displayed reduced worm burdens and liver pathology when compared to adjuvant controls. These findings demonstrate that a regulatory-compliant Sm29 antigen, combined with human-compatible adjuvants, induces robust immunity and protection against infection and supports further clinical advancement as a schistosomiasis vaccine candidate.

## Author summary

The helminth *Schistosoma mansoni* is one of the main causes of human schistosomiasis, a health and economic concern in some of the world's poorest countries. Current treatment regimens can lead to serious side effects and are not suitable for breastfeeding mothers. As such, efforts have been undertaken to develop a vaccine to prevent infection. The vaccine candidate tested in this study is the Sm29 protein present on the adult worm surface. One of the major challenges for vaccine development for humans based on recombinant proteins is the production of the molecule to reach high-quality antigen production and evaluation with clinically acceptable adjuvants. In this study, we produced the recombinant Sm29 in Good Laboratory Practice (GLP) and tested it formulated with alum or a squalene-based emulsion (CTVad1) in a murine model of *S. mansoni* infection. Both vaccine formulations induced a robust immune response and engendered partial protection against infection and reduced liver pathology. In summary, the Sm29 vaccine tested here showed reliability, quality and efficacy in pre-clinical studies before going to human trials.

## Introduction

Schistosomiasis is one of the most prevalent helminthic diseases worldwide, with major impacts on global health [1]. Infection occurs through host contact with cercariae, leading to skin penetration and adult worm maturation in the mesenteric veins. Following egg deposition, a large proportion of eggs become trapped in host organs such as the liver and intestine, where they trigger intense granulomatous inflammation and progressive fibrosis [2,3]. The granuloma is composed of macrophages, eosinophils, T and B lymphocytes, neutrophils, and fibroblasts, representing the main immunopathology of schistosomiasis [4,5].

Given the significant medical and socioeconomic impact of schistosomiasis, considerable efforts have been directed toward creating an effective vaccine to reduce transmission [6]. Developing a schistosomiasis vaccine is particularly challenging, as schistosomes possess a complex life cycle, multiple antigenic stages, and sophisticated immune evasion mechanisms [7,8]. Among the most promising vaccine candidates is the *Schistosoma mansoni* protein Sm29, identified through genomic approaches and recognized as a potent inducer of protective immunity [9,10]. In endemic regions, high levels of circulating anti-Sm29 IgG1 and IgG3 have been correlated with resistance to reinfection. Experimental models have demonstrated that immunization with recombinant Sm29 (rSm29) significantly reduces worm burden and induces high levels of IFN-γ and IgG1, indicating a mixed Th1/Th2 immune profile [11,12]. The protective potential of rSm29 highlights its relevance as a vaccine candidate, although the effectiveness of immunization depends largely on the adjuvant employed.

Adjuvants play a crucial role in vaccine formulations by enhancing antigen immunogenicity and modulating the immune response profile. Experimental studies using the *S. mansoni* protein Sm29 have often employed Freund’s adjuvant to enhance the immune response, and these studies demonstrated that rSm29 combined with Freund’s adjuvant can provide protection against reinfection in mice previously exposed to the parasite, inducing increased production of specific antibodies and a mixed cellular response, including elevated IFN-γ levels [11,12]. However, Freund’s adjuvant is not recommended for human use due to its high toxicity and the potential to induce severe inflammatory reactions [6]. Consequently, subsequent research has focused on safe adjuvants for human application, such as aluminum-based adjuvants and experimental formulations such as CTVad1 (squalene-based oil-in-water emulsion), aiming to develop effective and secure vaccines against schistosomiasis. Aluminum hydroxide (Alum) is one of the most widely used adjuvants in licensed human vaccines and is primarily associated with Th2-type humoral responses, although mixed profiles can occur depending on the antigenic formulation [13,14]. Previous studies have shown that rSm29 formulated with Alum elicits a strong antibody response and provides partial protection against *S. mansoni* reinfection [15]. Another adjuvant of particular interest is CTVad1, developed by the “Centro de Tecnologia de Vacinas” (Universidade Federal de Minas Gerais). Based on MF59, this squalene-based oil-in-water emulsion has demonstrated safety and efficacy in licensed human vaccines. CTVad1 was specifically designed to enhance both cellular and humoral immune responses, thereby potentially broadening the overall protective immune repertoire [16].

Ensuring antigen quality and safety is as crucial as optimizing adjuvants in vaccine development. The protective potential of rSm29 reinforces its relevance as a vaccine candidate and highlights the importance of producing recombinant proteins under standardized and controlled conditions. Adherence to Good Laboratory Practice (GLP) principles ensures reproducibility, regulatory compliance, and the reliability of preclinical data. Furthermore, recombinant proteins must meet stringent quality criteria regarding process-related impurities such as host cell proteins (HCPs), residual host cell DNA, endotoxins (LPS), and product integrity parameters, which are globally regulated to ensure product safety and efficacy [17–25]. This study evaluated the immunogenic potential of the recombinant Sm29 produced in Good Laboratory Practice and formulated with different adjuvants approved for human use according to regulatory agency requirements.

## Materials and methods

### Ethics statement

All experimental procedures involving animals were conducted in strict accordance with Brazilian legislation (Law No. 11794/2008) governing the use of animals in scientific research. The experimental protocol was reviewed and approved by the Committee on Ethics of Animal Experiments of the Federal University of Minas Gerais (UFMG) (Protocol Number 347/2025). Wild-type C57BL/6 mice were obtained from the Federal University of Minas Gerais (UFMG, Brazil). All animals were housed under specific-pathogen-free conditions at UFMG and used at 6–8 weeks of age. *S. mansoni* (LE strain) cercariae were provided by the Oswaldo Cruz Foundation – René Rachou Research Center (FIOCRUZ-CPqRR, Brazil) and obtained from infected *Biomphalaria glabrata* snails by exposure to light to induce parasite shedding.

### Sm29 gene design

The gene (based on Smp_072190) encoding the *S. mansoni* Sm29 antigen was synthetically produced and codon-optimized for expression in *Escherichia coli* by GenScript (Piscataway, NJ, USA) to maximize translational efficiency and protein yield (S1 Fig). The recombinant construct was cloned into the pET-24a expression vector under control of the T7 promoter. To enhance solubility and reduce aggregation, three additional aspartic acid residues were strategically introduced at the C-terminus of the protein sequence [26,27]. No polyhistidine (His-tag) or other affinity-purification tag was included in the design, enabling downstream applications that require tag-free protein formulations. The construct was sequence-verified, and chemically competent *SHuffle T7 E. coli* cells (New England Biolabs) were transformed via the standard heat shock method [28,29].

***In silico* physicochemical characterization.** The primary structure of the rSm29 recombinant construct (141 residues) was analyzed using sequence-based computational tools. Molecular weight, isoelectric point (pI), and net charge as a function of pH were calculated using standard amino acid residue masses and the Henderson–Hasselbalch equation with Lehninger pKa values. The molar extinction coefficient at 280 nm (ε_280_) was estimated according to [30]. Hydrophobicity was assessed using the Kyte–Doolittle scale [31] with a sliding window of 9 residues, and the protein stability index was computed according to [32]. All analyses were performed using custom Python scripts with NumPy and Matplotlib libraries.

### Expression, cell lysis, inclusion body washing steps, and solubilization

Recombinant Sm29 protein was expressed in *E. coli* and cultured in 1 L of Luria–Bertani (LB) medium under aerobic conditions. Induction was initiated at an optical density (OD₆₀₀) of 0.44 by adding isopropyl β-D-1-thiogalactopyranoside (IPTG) to a final concentration of 0.4 mM. Following induction, cultures were maintained at 20 °C for 18 h to promote protein expression. *E. coli* cells expressing rSm29 were harvested by centrifugation at 10000 × g for 10 minutes at 4 °C, resuspended in PBS pH 7.4 (Gibco), and supplemented with Phenylmethylsulfonyl fluoride (PMSF) 1mM (Sigma), Dithiothreitol (DTT) 5mM (Roche), and Benzamidine hydrochloride hydrate 5mM (Sigma). Cell pellets were subjected to mechanical disruption using a French Press (Brand) for 15 minutes to ensure complete lysis. Lysates were clarified by centrifugation at 40,000 × *g* for 30 minutes at 4 °C to separate the soluble fraction from insoluble inclusion bodies. The resulting pellet, enriched in inclusion bodies, was sequentially washed (three times) with wash buffer (100 mM Tris-HCl, 10 mM EDTA, 2 M urea, 10% glycerol, 2% Triton X-100, pH 7.0) to eliminate host-derived contaminants. A final wash was performed using phosphate-buffered saline (PBS, Gibco) to remove residual detergents before solubilization. Each wash step involved resuspension by pipetting, vortexing, and centrifugation at 40.000 × g for 30 minutes at 4 °C. Washed inclusion bodies were resuspended in denaturing buffer (20 mM sodium phosphate, 8 M urea, pH 6.7) and manually solubilized at room temperature through gentle pipetting and controlled homogenization. The solubilized protein solution was centrifuged at 40,000 × g for 30 minutes at 4 °C, and the supernatant was collected for a subsequent purification step.

### Purification by ion exchange chromatography and endotoxin removal

The clarified lysate was loaded onto a pre-equilibrated HiTrap Q XL anion exchange column (Cytiva) connected to an ÄKTA Pure system (Cytiva). The column was equilibrated with Buffer A (20 mM sodium phosphate, 8 M Urea, pH 6.7), and the protein sample was loaded at a flow rate of 5 mL/min. Unbound material was removed by washing with 10 column volumes of buffer A. Bound proteins were eluted using a linear gradient of NaCl (0-0.35 M) in Buffer B (20 mM sodium phosphate, 8M Urea, 1 M NaCl, pH 6.7) with 70 column volumes. Eluted fractions were monitored at 280 nm, collected, and analyzed by SDS-PAGE. Before additional chromatographic steps aimed at optimizing purification, the protein sample was dialyzed against Buffer A (20 mM sodium phosphate, 8 M urea, pH 6.7) to remove residual NaCl and equilibrate the ionic strength. An additional anion exchange chromatography was performed to further reduce contaminating host-derived proteins, following the same conditions as the initial purification, except for a modified elution profile. At this point in the chromatography workflow, Buffer A (20 mM sodium phosphate, 8 M urea, pH 6.7) was employed for column equilibration and washing. Specifically, the gradient of buffer B was adjusted to 0- 0.4 M NaCl, and the linear elution gradient was shortened from 70 to 30 column volumes (CV), thereby reducing the total elution volume required for protein recovery. Fractions were collected, analyzed by SDS-PAGE, and pooled based on purity. Following SDS-PAGE analysis, the protein concentration was determined using the Pierce BCA Protein Assay Kit (Thermo Fisher Scientific, Waltham, USA) according to the manufacturer’s instructions. Next, the protein sample was dialyzed overnight against an alternative Buffer A (50 mMacetic acid, 8 M urea, pH 4.3) to promote endotoxin (LPS) removal. Samples were dialyzed against 1 L of appropriate buffer at 4 °C using a Spectra/Por membrane (MWCO 6–8.000 kDa; Spectrum Medical Industries, Inc., Laguna Hills, CA), with gentle stirring using a magnetic stir bar. Subsequently, the dialyzed sample was applied to an anion exchange chromatography column for further purification. The rSm29 was applied to the column at a flow rate of 1 mL/min under these conditions and collected in the flow-through, as it does not bind significantly at this pH and conductivity. LPS molecules remained bound to the resin and were later removed during column regeneration. The flow-through fractions were pooled and quantified using Pierce BCA Protein Assay Kit (Thermo Fisher Scientific, Waltham, Massachusetts, USA), following the manufacturer’s protocol. Endotoxin removal efficiency was confirmed by the ToxinSensor Chromogenic LAL Endotoxin Assay Kit (GeneScript, Piscataway, NJ, USA), performed in triplicate. rSm29, initially solubilized in 8 M urea, was subjected to a dialysis protocol to reduce the urea concentration to 1 M, thereby equilibrating the sample in a buffer compatible with downstream applications. Dialysis was carried out at 4 °C under gentle agitation using a 10 kDa molecular weight cut-off (MWCO) membrane, against 1 L of buffer composed of 20 mM sodium phosphate, 1 M urea, and 150 mM NaCl at pH 7.4.

### Bacterial endotoxin quantification

Residual endotoxin quantification was performed at two distinct stages of the production process. For in-process control and rapid monitoring of the efficiency of LPS removal steps, the ToxinSensor Chromogenic LAL Endotoxin Assay Kit (GenScript, Piscataway, NJ, USA) was used according to the manufacturer's instructions.

For the final characterization of the Active Pharmaceutical Ingredient (API) and to ensure regulatory compliance, rigorous endotoxin quantification was conducted using the kinetic turbidimetric method, as recommended by the Pharmacopoeia. Briefly, samples were diluted in endotoxin-free water to prevent interference, as required to comply with the assay operating range. Potential matrix interference was assessed through the positive product control recovery according to the manufacturer's instructions.

The analysis was based on the reaction time required to reach a predetermined level of turbidity in the mixture of the sample and the Limulus Amebocyte Lysate (LAL) reagent. Concentrations were calculated by interpolation from a Reference Standard Endotoxin curve, and the results were expressed in Endotoxin Units per microgram of protein (EU/µg).

Acceptance criteria for residual endotoxins were established based on the maximum human dose and average body weight, as defined by pharmacopeial guidelines. The endotoxin limit (EL) was calculated using the formula EL = K/M, where K represents the threshold pyrogenic dose (5.0 EU/kg), and M is the maximum dose administered per kg per hour. Based on this calculation, the safety threshold for the rSm29 vaccine was defined as < 3.5 EU/µg, ensuring the product's safety for clinical use.

### SDS-PAGE and western blot analysis

The pooled fractions containing purified rSm29 were subjected to SDS-PAGE analysis and western blotting. Western blotting was employed to confirm the identity and immunoreactivity of the rSm29. Protein samples (0.1 to 0.5 µg) were resolved on a 15% SDS-PAGE gel as previously described by Laemmli [33] and electrotransferred to nitrocellulose membranes (Amersham Biosciences, Uppsala, Sweden) using a semidry system (Bio-Rad) at 25V/5mA for 25 minutes. Membranes were blocked for 1 hour at room temperature in TBS (Tris-buffered saline containing 0.05% Tween-20, pH 7.2), supplemented with 5% non-fat dry milk. Following the blocking step, membranes were incubated overnight at 4 °C with anti-Sm29 polyclonal antibodies (1:2000) previously produced in mouse. In the next step, membranes were washed three times with TBS-T and incubated for 1 hour at room temperature with goat anti-mouse IgG conjugated to horseradish peroxidase (HRP) (1:2000) - Invitrogen. Immunoreactive bands were detected using ECL western blotting detection reagents (Amersham Biosciences, Piscataway, USA) according to the manufacturer’s instructions and visualized on an Amersham Imager 600 (GE Healthcare).

### Circular dichroism spectroscopy and secondary structure analysis

Far-UV CD spectra were recorded on a Jasco spectropolarimeter (600 MHz, IME/EB) using a 0.1 mm pathlength quartz cuvette. The protein sample (rSm29, 0.8 mg/mL in 20 mM sodium phosphate, 1 M urea, 150 mM NaCl, pH 7.4) was measured in duplicate over the range 200–260 nm. Buffer baseline spectra were recorded under identical conditions using the same buffer diluted 10-fold and subtracted from the sample spectra prior to conversion to mean residue ellipticity (MRE). Spectra were smoothed using the Savitzky–Golay algorithm (window = 25 points, polynomial order = 3). The high tension (HT) voltage was monitored throughout acquisition as a criterion of signal reliability, with values below 600 V considered acceptable. Secondary structure content was estimated by non-negative least squares (NNLS) deconvolution using reference spectra for α-helix, β-sheet, and random coil derived from [34].

### Quantification of residual host cell DNA using a fluorescence-based assay

Residual double-stranded DNA (dsDNA) in protein samples was quantified using a high-sensitivity fluorescent assay (Quant-iT PicoGreen dsDNA Assay Kit, Thermo Fisher Scientific), following the manufacturer's protocol. Succinctly, the working reagent was freshly prepared by diluting the PicoGreendye 1:200 in 1 × TE buffer. In a black 96-well flat-bottom microplate (low-binding, fluorescence-compatible), 100 μL of each sample or DNA standard (λ DNA in TE buffer, provided in the kit) was pipetted in triplicate. An equal volume (100 μL) of PicoGreen working solution was added to each well, mixed gently, and incubated for 5 minutes at room temperature protected from light. Fluorescence was measured using a microplate reader (SpectraMax) with excitation at 480 nm and emission at 520 nm. A standard curve was generated using serial dilutions of λ DNA, and DNA concentrations in samples were interpolated using linear regression. Residual DNA concentrations were reported in ng per dose (based on previously determined protein concentration by BCA assay). Acceptance criteria followed regulatory guidelines for biopharmaceuticals, typically <10 ng/dose.

### Quantification of host cell proteins (HCP)

The presence of residual HCPs in purified recombinant protein samples was quantified using a commercial ELISA-based assay (Cygnus Technologies, USA), following the manufacturer’s instructions. Briefly, standards and appropriately diluted samples were added to 96-well microplates pre-coated with anti-HCPs polyclonal antibodies. After incubation, plates were washed to remove unbound material, and a horseradish peroxidase–conjugated secondary antibody specific for HCPs was added. Following a second wash step, the substrate solution (TMB) was added, and the reaction was allowed to develop for the recommended period. The enzymatic reaction was then stopped with acidic stop solution, and absorbance was measured at 450 nm with reference to 650 nm using a microplate reader. Residual HCPs were reported in ng per mg (based on previously determined protein concentration by BCA assay). Acceptance criteria followed regulatory guidelines for biopharmaceuticals, typically <100 ng/mg.

### Protein profiling using the bioanalyzer system

Protein sizing and purity assessment were performed using the Agilent 2100 Bioanalyzer system (Agilent Technologies, Santa Clara, CA, USA) in combination with the Protein 230 LabChip Kit, following the manufacturer’s instructions. Briefly, protein samples were heated at 95 °C for 5 minutes in a denaturing buffer containing SDS to achieve denaturation, enabling linearization and uniform charge-to-mass ratio of the polypeptides. Subsequently, 4 µL of each denatured sample was loaded into individual wells of the microfluidic chip, along with the appropriate molecular weight ladder and gel-dye mix. The chip was placed in the Bioanalyzer, where microfluidic capillary electrophoresis was automatically performed. Protein separation was based on size, under denaturing conditions, and fluorescence signals were detected and analyzed by the integrated software. Each electropherogram and virtual gel image was evaluated to determine the apparent molecular weights, relative abundance, and purity of each sample. The estimated molecular weights were calculated by comparison with the internal standard ladder, and peak areas were used for semi-quantitative assessment of relative protein concentration. The integrity of the proteins was confirmed by distinct, well-defined peaks at the expected molecular weights, with no evidence of smearing or degradation. Samples exhibiting multiple peaks or high-molecular-weight aggregates were flagged for potential contamination or incomplete denaturation.

All experimental activities were performed at CT Vacinas (Federal University of Minas Gerais, Brazil), a facility certified for Good Laboratory Practice (GLP/BPL) by the Brazilian National Institute of Metrology, Quality and Technology (INMETRO). Analytical assays and process development activities were conducted according to established standard operating procedures, ensuring traceability, quality control, and data integrity throughout the study.

### Mass spectrometry-based protein identity confirmation

Purified rSm29 was buffer-exchanged by methanol/chloroform precipitation and resuspended in 50 mM ammonium bicarbonate. Two aliquots of 20 µg were prepared for parallel digestion with trypsin or chymotrypsin (enzyme:protein ratio 1:50, w/w) after reduction with 10 mM DTT and alkylation with 40 mM iodoacetamide. Resulting peptides were desalted and analyzed by LC-MS/MS on a Vanquish Neo system coupled to an Orbitrap Ascend Tribrid mass spectrometer (Thermo Scientific), using a reverse-phase Acclaim PepMap column (50 cm × 75 µm, 2 µm) with an acetonitrile/formic acid gradient at 300 nL/min. MS1 and MS2 spectra were acquired in data-dependent mode (DDA) at resolving powers of 60,000 and 15,000, respectively, with HCD fragmentation at 25% normalized collision energy. For protein identity confirmation, raw data were processed in Proteome Discoverer 3.2 (Sequest HT) against a database containing the rSm29 sequence and common contaminants (precursor tolerance: 10 ppm; fragment tolerance: 0.02 Da; fixed modification: carbamidomethyl-Cys; up to 4 missed cleavages; FDR 1%). All PSMs were manually curated.

### Mice immunization protocol

C57BL/6 mice (6–8 weeks old) were housed under specific pathogen-free (SPF) conditions and handled following institutional animal care and guidelines. Mice were randomly assigned to experimental and control groups (n = 10 per group). Immunizations were performed subcutaneously, at the nape of the neck, using 25 μg of rSm29 formulated with Alum or CTVad1 (vol/vol). Control animals were injected with adjuvant alone at the same volume and on the same schedule. Mice received three doses at 14-day intervals (Days 0, 14, and 28). Sera were collected by submandibular bleeding at baseline (pre-immune) and two weeks after each immunization.

### Specifics antibodies detection by indirect ELISA

Total IgG, IgG1, IgG2c, IgG3 and IgE antibody responses in mouse sera were quantified by indirect ELISA. Following immunization, sera were collected from mice in each experimental group at 2-week intervals. High-binding microplates (Nunc MaxiSorp, Thermo Fisher Scientific) were coated overnight at 4 °C with 100μL/well of rSm29 (10μg/mL) diluted in carbonate-bicarbonate buffer (pH 9.6). Plates were washed three times with PBS containing 0.05% Tween-20 (PBST) and blocked with 100μL/well of 5% BSA in PBS for 1 hour at room temperature (11). After blocking, serum samples diluted 1:100 in PBST containing 5% BSA were added in triplicate and incubated for 2 hours at room temperature. To determine total IgG antibody titers, pooled serum samples from each group were used in a serial dilution range from 1:20–1:1,310,720 [12]. After washing, plates were incubated for 1 hour with horseradish peroxidase (HRP)-conjugated goat anti-mouse antibodies specific for IgG (1:5,000), IgG1 (1:10,000), IgG2c (1:12,000), IgG3 (1:2.000) and IgE (1:2.000) (Southern Biotechnology, CA, USA) [35]. The enzymatic reaction was developed by adding 100μL/well of TMB substrate for 15min, stopped with sulfuric acid (H_2_SO_4_), and absorbance was measured at 450 nm using a microplate reader (BioRad, Hercules, CA, USA).

### Cercarial challenge and parasite burden evaluation

Following standard procedures, mice were percutaneously challenged with *S. mansoni* cercariae (LE strain) two weeks after the final immunization. Briefly, six-to-eight-week-old wild-type mice were anesthetized with 5% ketamine, 2% xylazine, and 0.9% NaCl. Animals were individually exposed to 100 viable cercariae through the shaved abdominal skin for 1 hour using the cover slip technique. Cercarial viability and concentration were confirmed microscopically before infection. Forty-five days after challenge, mice were euthanized, and the portal and mesenteric veins were perfused with pre-warmed citrate-saline solution (0.85% NaCl, 1.5% sodium citrate) to recover adult worms [36]. The perfusate was passed through a 100 μm mesh and examined under a stereomicroscope. Worms were counted manually and classified by sex. Total worm burden per animal was recorded, and the protection level was calculated by comparing immunized groups to the control group [35].

### Splenocyte culture and cytokine determination

Cytokine secretion determination was conducted using splenocyte cultures from individually immunized mice (n = 5 per group). Splenocytes were isolated from macerated spleens of individual mice 15 days after the third immunization and washed twice with sterile PBS. After washing, cells were adjusted to 1 × 10^5^ cells per in wells RPMI 1640 medium (Gibco, CA, USA) supplemented with 10% fetal bovine serum (FBS), 100 U/mL sodium penicillin G, 100 μg/mL streptomycin sulfate, and 250 ng/mL amphotericin B. Splenocytes were cultured in medium alone or stimulated with rSm29 (25 μg/mL) or concanavalin A (ConA) (5 μg/mL), as previously described (11). Cells were incubated at 37 °C with 5% CO_2_. Culture supernatants were collected after 48 hours for IL-4 measurements, and after 72 hours for IFN-γ and IL-10 measurements by ELISA (R&D Diagnostics, Minneapolis, MN, USA) according to the manufacturer’s instructions.

### Liver pathology

After euthanasia, the left liver lobe was fixed in 10% buffered formaldehyde in PBS. Histological sections were prepared at 6 μm using a microtome and stained with Hematoxylin-Eosin (HE). For granuloma size, images were captured using a Michrome 20 digital camera coupled to a microscope (20 × objective) and the Mosaic 3.0 software (Tucsen Imaging Technology Co., Ltd). Analyses were performed using ImageJ software (National Institutes of Health, Bethesda, MD, USA). Granuloma size was measured in mm^2^ for all granulomas observed in liver sections. The number of eggs per granuloma was also counted.

### Statistical analysis

Data normality was tested using D’Agostino-Pearson omnibus tests. Statistical analyses were performed using ANOVA and Dunn’s multiple comparison test for the antibody measurements. The percentage of granuloma/liver areas was plotted in box plots (median, maximum, and minimum) and analyzed via the Kruskal-Wallis test followed by Dunn’s multiple comparison test. The number of granulomas per liver area and the number of eggs per granuloma were evaluated using one-way ANOVA followed by Tukey's multiple comparison test. The level of protection in infected mice was determined using Student’s *t*-test. All calculations were performed using GraphPad Prism 6 (GraphPad Software, La Jolla, CA, United States). The *p*-values were considered significant when < 0.05.

## Results

### Design and *in silico* characterization of the optimized Sm29 construct

The gene encoding the Sm29 target protein was successfully cloned into the pET24a(+) expression vector under the control of the T7 promoter (GenScript), enabling high-level, inducible expression in the *E. coli* Shuffle strain. Codon optimization was performed to enhance translational efficiency in *E. coli*, by reducing the frequency of rare codons and maximizing predicted mRNA stability without altering the amino acid sequence. The expression cassette was engineered without affinity tags to comply with regulatory requirements and preserve the native biochemical and immunological properties of the protein. The final construct therefore expresses the recombinant protein in its native form, without a C-terminal or N-terminal His-tag or other fusion domains, ensuring compatibility with pre-clinical development and analytical characterization workflows. To enhance solubility during protein expression in *E. coli*, three aspartic acid residues (DDD) were rationally introduced at the C-terminus of the protein (S1 Fig). Incorporation of negatively charged residues at the C-terminus has been associated with reduced aggregation propensity and enhanced solubility through increased electrostatic repulsion and disruption of hydrophobic aggregation interfaces. *In silico* solubility prediction demonstrated an increase in solubility scores relative to the native sequence, supporting this engineering strategy [37–40].

Sequence confirmation by Sanger sequencing validated the integrity of the transgene and the successful integration of the DDD motif. Alignment of sequencing chromatograms with the reference plasmid map demonstrated 100% identity with the designed construct, with no frameshift mutations, premature stop codons or unexpected sequence alterations. Restriction digestion analysis using *NdeI* and *XhoI* yielded the expected fragment pattern, further confirming proper assembly of the recombinant plasmid. Additionally, the recombinant plasmid was confirmed to maintain the kanamycin resistance cassette and origin of replication associated with pET-24a (+), ensuring stable selection during bacterial amplification and protein expression. The final construct was designated pET-24a-Sm29-DDD. This native recombinant protein expression construct incorporating a C-terminal DDD solubility enhancement motif was successfully engineered, sequence-validated, and was suitable for downstream expression, purification, and pre-clinical quality assessment.

The primary structure analysis of Sm29 reveals several physicochemical features consistent with its classification as a member of the Ly6/uPAR (three-finger fold) protein family [41]. The recombinant construct comprises 141 amino acid residues with a calculated molecular weight of 15.39 kDa and an isoelectric point (pI) of 4.66, conferring a net negative charge of −5.6 at physiological pH (7.4), which is consistent with the anion-exchange chromatography purification strategy employed. The most striking feature of the primary structure is the exceptionally high cysteine content — 17 residues comprising 12.1% of the sequence — arranged in a characteristic spacing pattern (CxxC and CxxxC motifs) that supports the formation of up to eight disulfide bonds, the hallmark of the Ly6/uPAR LU domain [42,43]Notably, the protein lacks tryptophan residues entirely, resulting in a relatively low molar extinction coefficient at 280 nm (ε_280_ = 8,940–9,940 M^−1^cm^−1^, depending on the oxidation state of cysteine residues), calculated according to [30]. The overall hydrophilicity (GRAVY index = −0.119) is consistent with a surface-exposed protein, and the computed instability index of 21.77 predicts conformational stability in solution, likely stabilized by the extensive disulfide bond network. The C-terminal DDD motif, rationally introduced to enhance solubility during recombinant expression in *E. coli*, contributes three additional aspartate residues to the overall negative charge of the protein at physiological pH (S1 Fig).

### Anion exchange chromatography purification strategy

**Fig 1:**
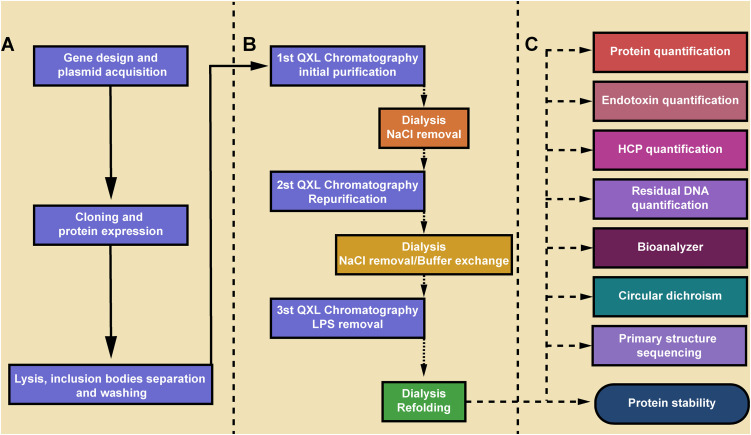
Workflow diagram of the expression, purification process, and sample quality control steps for Sm29 recombinant protein. Schematic representation of the production process of recombinant Sm29 expressed in *Escherichia coli*, including bacterial cultivation, cell disruption by high-pressure homogenization, clarification of the soluble and insoluble fractions, recovery of inclusion bodies, solubilization under denaturing conditions, and sequential purification by anion-exchange chromatography. The downstream process comprised an initial capture step, an intermediate purification step to reduce host cell-derived contaminants, and a polishing step dedicated to endotoxin removal, followed by gradual reduction of the chaotropic agent by stepwise dialysis and formulation in the final storage buffer. Analytical characterization included determination of protein concentration by BCA assay, assessment of purity by SDS-PAGE and microfluidic electrophoresis, immunoreactivity by Western blot and ELISA, quantification of residual host-cell proteins (HCPs), residual host-cell DNA, and endotoxin levels. Each purification stage was designed to progressively reduce process-related impurities while maximizing recombinant protein recovery and maintaining product quality attributes relevant to future vaccine development.

All stages of the rSm29 expression and optimization process are described in detail in Fig 1A-1C. The purification of the rSm29 was conducted using an anion exchange chromatography strategy employing a QXL column functionalized with quaternary amine ligands. This resin provides a positively charged matrix that selectively binds negatively charged biomolecules under controlled ionic conditions. The first QXL chromatography step was primarily designed as a capture step for bulk impurity reduction and target protein enrichment.

**Fig 2:**
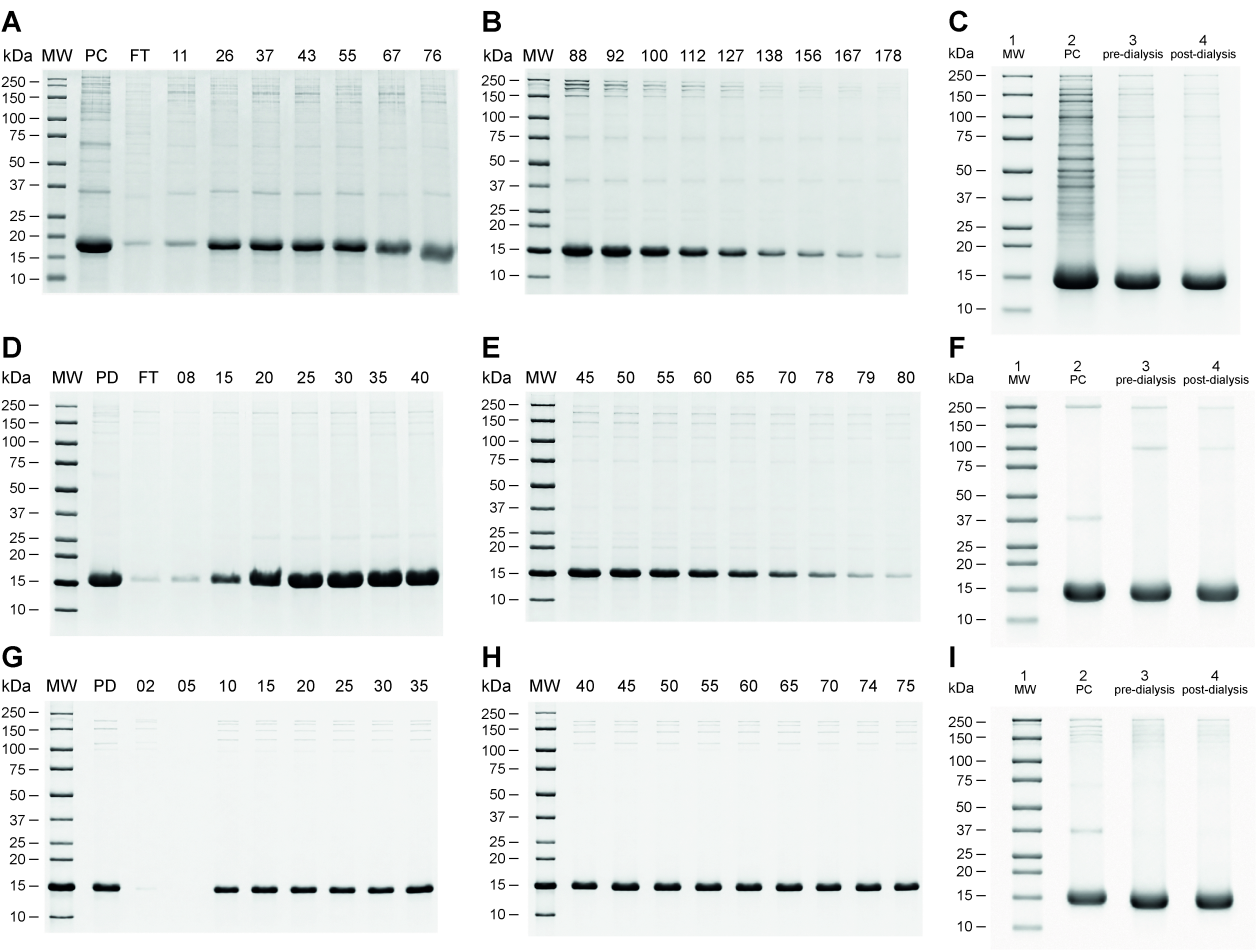
SDS-PAGE analysis of chromatographic fractions obtained during recombinant Sm29 purification. A and B- SDS-PAGE (15%) analysis of fractions obtained during the purification of 80 mL of solubilized recombinant Sm29. MW: molecular weight marker (kDa); PC: solubilized sample prior to column loading; FT: flow-through fraction; lanes 11 to 178 correspond to 2-mL fractions collected during the purification process. A total of 30 μL of each sample was loaded per lane. C- SDS-PAGE (15%) analysis of pooled recombinant Sm29 fractions (55 to 126) before and after dialysis. The predominant protein band migrated at the expected molecular weight of approximately 15.34 kDa, indicating enrichment of recombinant Sm29 and preservation of protein purity throughout the dialysis process. D and E- Electrophoretic profile of fractions collected during the repurification of Sm29. MW: molecular weight marker (kDa); PC: sample prior to column loading; FT: flow-through fraction; lanes 08 to 80 correspond to 2-mL fractions collected during the repurification process. F- SDS-PAGE (15%) analysis of pooled recombinant Sm29 fractions (09 a 78) before and after dialysis. A total of 30 μL of each sample was loaded per lane. G and H- Electrophoretic profile of fractions collected during the endotoxin removal purification. MW: molecular weight marker (kDa); PC: sample prior to column loading; lanes 02–75 correspond to sequential 2-mL fractions collected during the purification process. I- Electrophoretic profile of fractions collected during the endotoxin removal purification. MW: molecular weight marker (kDa); PC: sample prior to column loading; lanes 10 a 75 correspond to sequential 2-mL fractions collected during the purification process. A total of 30 μL of each sample was loaded per lane. *Pre-dialysis: pooled elution fractions prior to buffer exchange; post-dialysis: pooled fractions following dialysis.

Following the first purification step, 15% SDS-PAGE analysis was performed to evaluate the expression profile and purity of the recombinant Sm29 protein (Fig 2). Based on the electrophoretic profiles (11 a 178) obtained from the loaded fractions (30 μL per lane), purified fractions exhibiting the highest purity (Fig 2A and 2B) and recovery were pooled for further processing (Fig 2C). The total volume of the pooled fractions was recorded before dialysis. Fractions 55–126 were combined, resulting in a final pool volume of 142 mL. To remove residual salt and prepare the sample for the subsequent repurification step aimed at further reducing host cell-derived contaminants, the pooled protein solution was dialyzed overnight against 20 mM sodium phosphate buffer containing 8 M urea (pH 6.7). After dialysis, the pool volume remained unchanged at 142 mL, indicating that no significant sample loss occurred during buffer exchange, at a concentration of 0.74 mg/mL, corresponding to a total protein recovery of 105.1 mg of rSm29.

The purification process was performed at pH 6.7, a value above the theoretical isoelectric point (pI = 4.96) of rSm29. Under these conditions, the recombinant protein carried an overall negative net charge, enabling binding to the anion-exchange matrix. Consistent with the chromatographic behavior observed during process optimization, this interaction was sufficient to retain the protein on the column but was readily disrupted by relatively low NaCl concentrations during elution.

The second QXL step functioned as an intermediate purification stage, improving protein purity and reducing residual host-cell contaminants. During the loading phase, the target protein was adsorbed onto the resin while unbound impurities were removed in the flow-through Controlled elution was achieved by gradually increasing the ionic strength through a NaCl gradient, which weakened the electrostatic interactions and promoted desorption of the protein. This purification approach provided effective removal of HCPs and other process-related impurities, while maintaining structural integrity and activity of the recombinant protein. The use of a QXL strong anion exchanger at optimized pH conditions has been widely reported as a robust and scalable method for downstream purification of acidic recombinant proteins, ensuring both high recovery and regulatory-grade purity.

For the second chromatography step (intermediate purification), the entire dialyzed pool (144 mL) was loaded onto the QXL column. Based on the SDS-PAGE profiles of the collected fractions (08–80) (Fig 2D and 2E) and fractions 09–78 were selected and combined (Fig 2F). After the intermediate purification step, the pooled fractions contained 140 mL at 0.77 mg/mL, yielding 107.8 mg of recombinant protein. Before the endotoxin removal chromatography step, the pooled sample (140 mL) was dialyzed overnight against 50 mM acetic acid buffer containing 8 M urea (pH 4.3) to perform buffer exchange and prepare the sample for the subsequent purification stage. Following dialysis, the pool volume increased from 140 mL to 147 mL. This increase is consistent with osmotic effects arising from differences in buffer composition and ionic properties between the sodium phosphate and acetic acid dialysis buffers. The lower ionic strength and altered physicochemical characteristics of the acetic acid buffer may have promoted water influx across the dialysis membrane, resulting in a modest increase in sample volume. Despite this volumetric variation, no visible protein precipitation was observed, and the sample remained suitable for the subsequent chromatography step.

Additionally, a third anion exchange chromatography strategy was employed using a QXL strong anion exchange column to efficiently remove endotoxin contaminants from the recombinant protein preparation. The third QXL chromatography step acted as a polishing stage specifically optimized for lipopolysaccharide (LPS) reduction and final impurity clearance.

The QXL resin provides a positively charged matrix that binds negatively charged molecules, such as lipopolysaccharides (LPS). Purification was performed at pH 4.3, a condition in which the LPS molecules (pI ≈ 2.0) carry a strong negative charge, thereby exhibiting high affinity for the positively charged resin. In contrast, the target protein rSm29, with a theoretical isoelectric point (pI = 4.96), remains near its net neutral charge under these conditions and consequently displays minimal interaction with the matrix. As a result, the protein of interest was efficiently recovered in the flow-through fraction, while endotoxins were retained on the column This selective separation mechanism effectively reduced endotoxin burden to levels compliant with pharmacopeial and regulatory limits for parenteral biologics. The approach demonstrates a practical application of charge-based differential binding for endotoxin clearance, providing a scalable, non-denaturing purification step that is compatible with GLP bioprocessing standards.

For the anion-exchange chromatography step dedicated to endotoxin (LPS) removal, 147 mL of the Sm29-containing pool was loaded onto the QXL column. Under these conditions, the recombinant protein did not bind to the chromatographic matrix, and the entire flow-through fraction was collected individually throughout the purification process. Following sample collection, the chromatographic run was completed, and the endotoxin content of the recovered fractions was subsequently determined. Fraction quality and protein integrity were evaluated (02–75) (Fig 2G and 2H) by 15% SDS-PAGE analysis, which was used to identify the fractions containing the target protein and assess the effectiveness of the purification strategy (10–75) (Fig 2I). Following the endotoxin removal step, the final pool consisted of 104 mL at 0,88 mg/mL, corresponding to 91,5 mg of purified rSm29.

Throughout the purification process, 8 M urea was maintained during all three anion-exchange chromatography steps and during the first two intermediate dialysis procedures. This strategy was adopted to preserve Sm29 in a soluble state after recovery from inclusion bodies and to minimize protein aggregation and precipitation during purification. Because Sm29 contains multiple cysteine residues and was initially solubilized under strongly denaturing conditions, premature removal of urea could promote intermolecular interactions and irreversible aggregation, leading to product loss and reduced process robustness. Therefore, urea concentration was reduced gradually only during the final refolding stage through sequential dialysis steps, allowing controlled protein renaturation while maintaining high recovery yields.

Following the final dialysis step in buffer containing 1 M urea, the final preparation volume was 100 mL with a protein concentration of 1.06 mg/mL, as determined by the BCA assay. This corresponded to a total recovery of approximately 100 mg of purified rSm29, yielding approximately 100 mg of recombinant protein per liter of bacterial culture. The overall protein recovery throughout the purification process remained above 98%, indicating that the sequential chromatographic steps did not significantly compromise protein yield.

### Recombinant protein yield, purity, and identification by SDS-PAGE and western blot

The recombinant protein was successfully expressed and purified under optimized induction and downstream conditions, yielding consistent production across independent fermentation batches. From a 1 L bacterial culture, the final recovery of purified target protein averaged 100 mg (mean ± SD, n = 3), as determined using the BCA assay. Process reproducibility was demonstrated by low inter-batch variation (<10%), indicating robustness of the expression and purification strategy. The achieved yield aligns with the expected productivity range for soluble recombinant proteins expressed in *E. coli* under standard laboratory-scale fermentation parameters (0.1-5 g/L, depending on construct and solubility tag) [44–46]. This performance reflects the effectiveness of vector design and culture optimization parameters, particularly induction temperature, IPTG concentration, and buffer composition, which collectively enhanced solubility and minimized inclusion body formation. The obtained yield is suitable for subsequent analytical characterization, pre-formulation, and preclinical testing stages of the active pharmaceutical ingredient (API) development.

Pooled fractions obtained after purification steps were analyzed by SDS-PAGE to evaluate protein purity and recovery. The electrophoretic profile revealed a predominant band at the expected molecular mass of approximately 15 kDa, corresponding to the recombinant protein of interest, with minimal detectable impurities after each purification step (Fig 2C, 2F and 2I).

**Fig 3:**
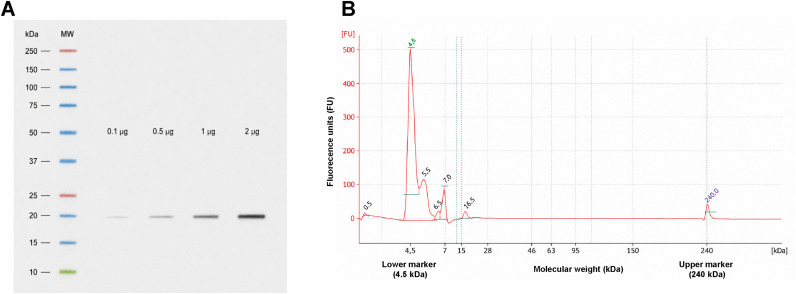
Sm29 recombinant protein Western blotting and Bioanalyzer. (A) Western blot analysis of recombinant Sm29 loaded at different concentrations (0.1–0.5 μg). An immunoreactive band at the expected molecular weight of approximately 15.34 kDa was observed across all protein loads, confirming the detection of recombinant Sm29 by the anti-Sm29 antibody. (B) Microfluidic electrophoresis analysis of recombinant Sm29 using the Agilent 2100 Bioanalyzer. The electropherogram and corresponding virtual gel revealed a predominant protein species with an apparent molecular weight of approximately 15.34 kDa, consistent with the expected size of recombinant Sm29. Quantitative analysis indicated an estimated protein purity of approximately 95%, supporting the effectiveness of the purification process and the enrichment of the target protein.

Densitometric analysis of SDS-PAGE profiles demonstrated a high degree of sample purity both before and after dialysis, with no detectable degradation or formation of secondary bands, indicating structural stability of the recombinant protein. Comparative quantification revealed consistent band intensity corresponding to the target molecular weight, confirming that the dialysis process did not lead to significant protein loss or aggregation. The calculated purity exceeded 95%, consistent with preparative chromatography performance and appropriate buffer exchange conditions (Fig 3B). Furthermore, the overall yield after dialysis remained above 98% of the pre-dialysis protein concentration, supporting the robustness of the purification and formulation procedures in maintaining protein integrity and recovery. These results confirm that the selected dialysis parameters ensured efficient removal of low-molecular-weight impurities while preserving protein stability and purity in accordance with regulatory expectations for preclinical-grade biopharmaceutical materials.

Protein identity was further confirmed by Western blotting using a specific polyclonal antibody raised against the recombinant Sm29 (Fig 3A). A single immunoreactive band was detected at the same molecular weight observed by SDS-PAGE, confirming antigenic integrity and absence of degradation products or cross-reactive impurities. Together, these results validate the effectiveness of the purification strategy in producing a homogeneous and immunoreactive recombinant protein suitable for analytical characterization, formulation, and preclinical evaluation.

### Analytical strategies for detection and quantification of process-related impurities during recombinant protein production

The analytical characterization of process-related impurities is a critical component of the quality assessment of recombinant protein products. These impurities, originating from the host cell or manufacturing process, included host cell proteins (HCPs), residual host cell DNA, endotoxins, and protein integrity, which were assessed by microfluidic electrophoresis. Their identification and quantification ensure that the API meets safety and purity criteria established by international regulatory guidelines. Due to the relatively weak interaction of recombinant Sm29 with the anion-exchange matrix under the selected operating conditions, the presence of NaCl in the washing buffer promoted premature target protein desorption. Therefore, a salt-free washing step was adopted to preserve product recovery while maintaining satisfactory impurity removal.

### Host cell proteins (HCPs)

The levels of HCPs were quantified using a validated ELISA specific for the *E. coli* expression system. Across production batches, there was consistent HCP clearance throughout downstream purification. Initial harvest material contained elevated concentrations of HCPs, which were progressively reduced following chromatographic purification.

The HCP content measured for the rSm29 protein was 140 ng/mg (Table 1). While this value is near the established target of ≤100 ng/mg, it remains within a range consistently associated with high-purity biologicals and aligns with levels often accepted in early-stage vaccine development. The observed consistency across multiple batches demonstrates the efficiency of the downstream process in managing host cell impurities. Moreover, this level of residual HCP did not interfere with the protein's safety profile in the subsequent preclinical evaluations, confirming the suitability of the purified rSm29 for its intended applications.

**Table 1:** Analytical characterization and quality attributes of the purified rSm29 protein.

Parameter	Analytical Method	Quality Attributes Target	Results Obtained
Protein Purity	Microfluidic electrophoresis (Bioanalyser)	> 80%	95% (Main peak)
Host Cell Proteins (HCP)	ELISA (anti-host cell antibodies)	≤ 100 ng/mg of protein*	140 ng/mg of protein
Endotoxins	kinetic turbidimetric method with LAL reagent	≤ 3.5 EU/µg of protein	0.154 EU/µg of protein
Residual Host Cell DNA	High-sensitivity fluorescent assay	≤ 10 ng/dose	≤ 3.5 ng/dose

*Target level defined for high-purity recombinant antigens; values slightly above the target were considered acceptable based on the consistent safety profile in preclinical assays.

### Residual DNA

Residual host cell DNA content was evaluated using a high-sensitivity fluorescent assay (Quant-iT PicoGreen dsDNA Assay Kit, Thermo Fisher Scientific). The measured concentration of residual DNA in the final purified recombinant protein preparation was 3.5 ng DNA per therapeutic dose as demonstrated in Table 1. This value is well below the international regulatory limit of <10 ng DNA per therapeutic dose, as established by the World Health Organization [47] and ANVISA [21] for recombinant biopharmaceuticals. These results confirm that the purification process efficiently removed host-derived nucleic acids, ensuring compliance with global quality and safety standards for preclinical and toxicological applications.

### Endotoxin quantification

Endotoxin concentrations were measured by the kinetic turbidimetric method using the Limulus Amebocyte Lysate (LAL) reagent. Endotoxin analysis performed after the second QXL chromatography revealed a residual endotoxin concentration of 58 EU/mg of protein. This result demonstrated that, despite the effectiveness of the capture and intermediate purification steps, additional endotoxin clearance was required. Consequently, a third QXL chromatography step was introduced as a polishing stage specifically aimed at reducing endotoxin levels. The substantial reduction achieved after this final chromatographic step confirmed the necessity and effectiveness of this additional purification stage. Process intermediates demonstrated a marked decrease in endotoxin levels. Final endotoxin concentrations were consistently below 0.154 EU/µg total protein (Table 1), well within the specific safety limit calculated for rSm29 (3.5 EU/µg total protein). The observed results indicate adequate control of upstream bacterial lysis and effective downstream endotoxin removal capability. The validity of the LAL assay was confirmed through the acceptance criteria of the positive product control, indicating that residual buffer components, including urea, did not significantly interfere with endotoxin quantification.

### Bioanalyzer protein integrity profile

Microfluidic electrophoresis (Bioanalyzer) confirmed protein purity and structural integrity at critical stages of manufacturing. The purified final product displayed a predominant band at the expected molecular weight (15 kDa), with purity exceeding 95% (Table 1), as determined by peak area integration of the main protein peak. No fragmentation or aggregation species were detected. Batch-to-batch profiles were highly comparable, supporting manufacturing consistency. Absence of degradation species indicates effective protein stabilization, appropriate chromatographic selection, and consistent product quality (Fig 3B).

### Mass spectrometry-based identity confirmation of rSm29

**Fig 4:**
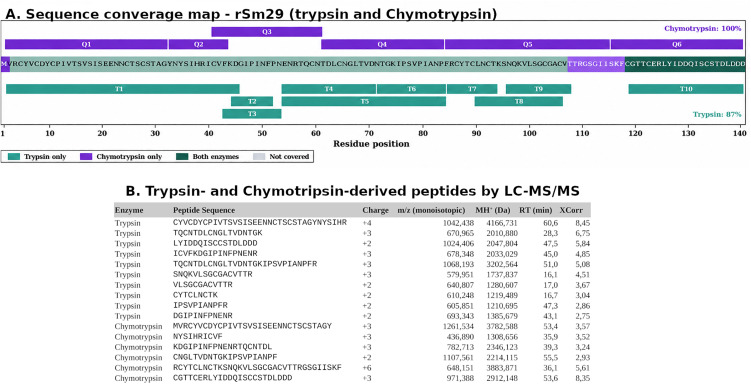
LC-MS/MS-based sequence coverage and identity confirmation of rSm29. **(A)** Peptide coverage map of the 141-amino acid rSm29 recombinant construct obtained by LC-MS/MS following enzymatic digestion with trypsin and chymotrypsin. Each residue is color-coded according to the enzyme generating the covering peptide: teal, trypsin only; purple, chymotrypsin only; dark green, covered by both enzymes; light grey, not covered. Trypsin-derived peptides (T1–T10, bars below the sequence) achieved 87% sequence coverage, chymotrypsin-derived peptides (Q1–Q6, bars above the sequence) achieved 100% coverage, and the combined dual-enzyme strategy yielded complete 100% coverage of the full-length construct including the engineered C-terminal DDD motif. **(B)** Experimental parameters of trypsin- and Chymotripsin-derived peptides identified by LC-MS/MS: peptide sequences are reported with carbamidomethylated cysteines (+57.021 Da); charge state, monoisotopic m/z, protonated molecular mass (MH⁺), chromatographic retention time (RT), and Sequest HT XCorr score are shown for each peptide. All peptides were detected at MS1 and MS2 levels and manually curated. Data were acquired on an Orbitrap Ascend Tribrid mass spectrometer and processed with Proteome Discoverer 3.2 (precursor tolerance: 10 ppm; fragment tolerance: 0.02 Da; FDR: 1%).

To unambiguously confirm the identity and assess the protein composition of the purified rSm29 preparation, the final product was subjected to LC-MS/MS analysis following proteolytic digestion with two complementary enzymes, trypsin and chymotrypsin (Fig 4A). This orthogonal dual-enzyme strategy was used to maximize sequence coverage and ensure that all regions of the recombinant construct, including cysteine-rich segments and regions refractory to a single protease, were represented in the peptide map.

Trypsin digestion generated ten peptides (T1–T10) covering 87% of the rSm29 sequence, while chymotrypsin digestion generated six peptides (Q1–Q6) independently achieving 100% coverage (Fig 4B). The combined analysis yielded complete 100% sequence coverage of the 141-amino acid construct, including the engineered C-terminal DDD motif (Fig 4). All identified peptides were detected at both MS1 and MS2 levels and manually curated, with XCorr scores ranging from 2.75 to 8.45 for tryptic peptides and 2.93 to 8.35 for chymotryptic peptides, reflecting high-confidence spectral matches. The complete sequence identity of the recombinant protein was thus confirmed at the peptide level, providing stronger evidence of protein identity than immunoblotting alone.

### Circular dichroism reveals a three-finger fold spectral signature in refolded rSm29

**Fig 5:**
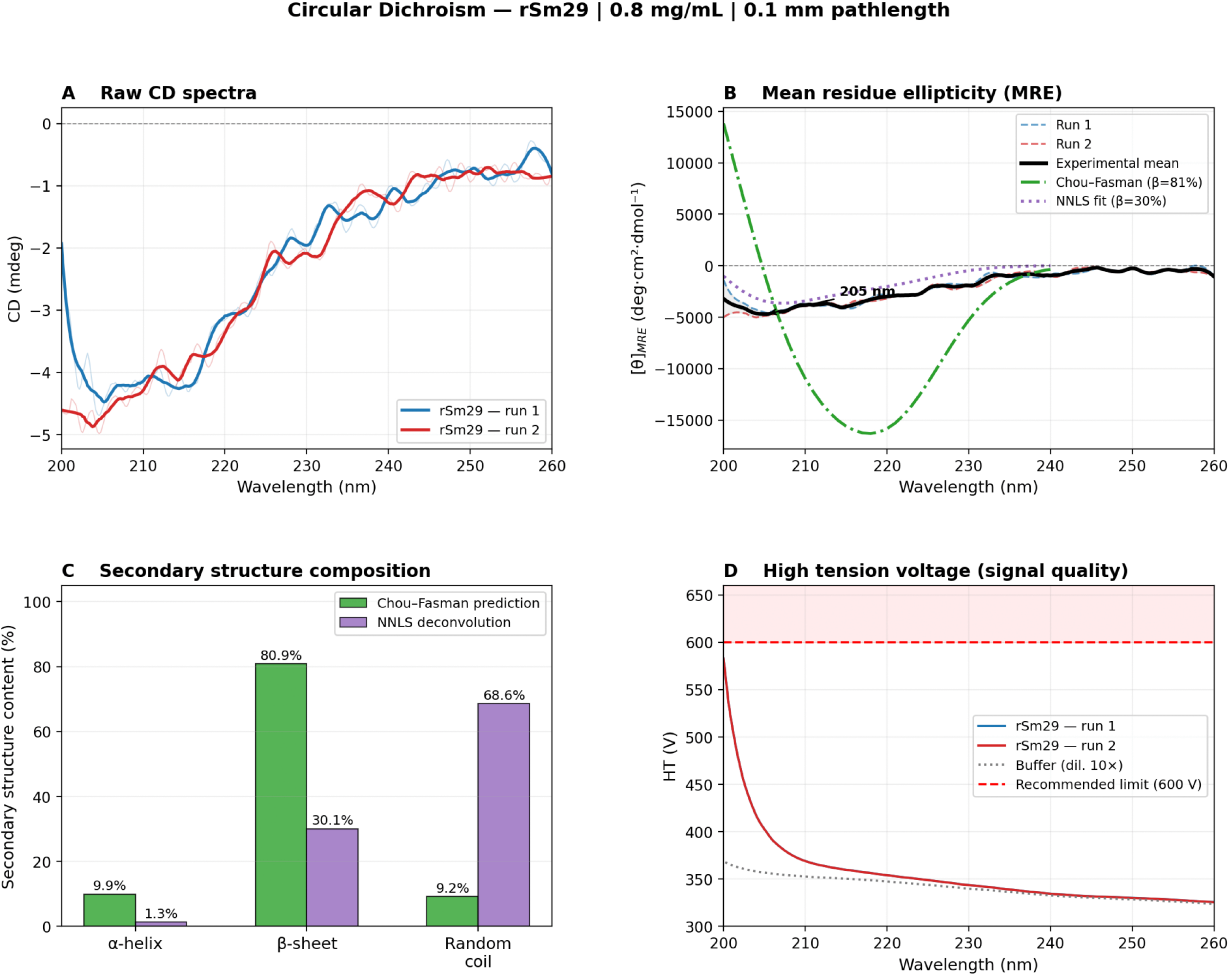
Circular dichroism analysis of recombinant Sm29. (A) Raw circular dichroism (CD) spectra of recombinant Sm29 obtained from two independent measurements, showing the reproducibility of the spectral profile between runs. **(B)** Mean residue ellipticity (MRE) spectra calculated from the experimental data and compared with the Chou-Fasman secondary structure prediction and the non-negative least squares (NNLS) deconvolution fit. The experimental spectrum exhibited a minimum around 205 nm, suggestive of the presence of mixed secondary structural elements. **(C)** Secondary structure composition estimated from the amino acid sequence using the Chou-Fasman algorithm and from CD spectral deconvolution by the NNLS method, showing the relative contributions of α-helices, β-sheets, and random coil structures. **(D)** High-tension (HT) voltage profiles recorded during CD measurements, demonstrating that the signal quality remained within the recommended instrumental limits (<600 V) throughout the analyzed spectral range. CD measurements were performed using recombinant Sm29 at a concentration of 0.8 mg/mL in a 0.1-mm pathlength quartz cuvette.

The far-UV CD spectrum of rSm29 displays a broad negative band centered at ~205 nm. The signal-to-noise ratio at lower wavelengths is partially affected by the buffer composition, which contains residual urea and salt that absorb in the far-UV region; however, the high tension (HT) voltage remained below 600 V throughout the entire spectral range (200–260 nm), confirming that the acquired signal is reliable and that the data are within acceptable quality thresholds for spectral interpretation [48]. This spectral profile is consistent with the structural characteristics of Ly6/uPAR (three-finger fold) family members, which are dominated by short β-strands, abundant β-turns, and loops projecting from a disulfide-stabilized core [42,43]. In such proteins, the classical β-sheet minimum at ~217 nm is shifted and broadened due to contributions from β-turns and disulfide bonds to the far-UV CD signal [49,50], resulting in a spectral envelope distinct from canonical antiparallel β-sheet proteins. These data provide experimental evidence that the refolding process from inclusion bodies yielded a structurally competent rSm29 protein [48] (Fig 5A-5D).

### Specific humoral response in mice after immunization with rSm29 formulations

**Fig 6:**
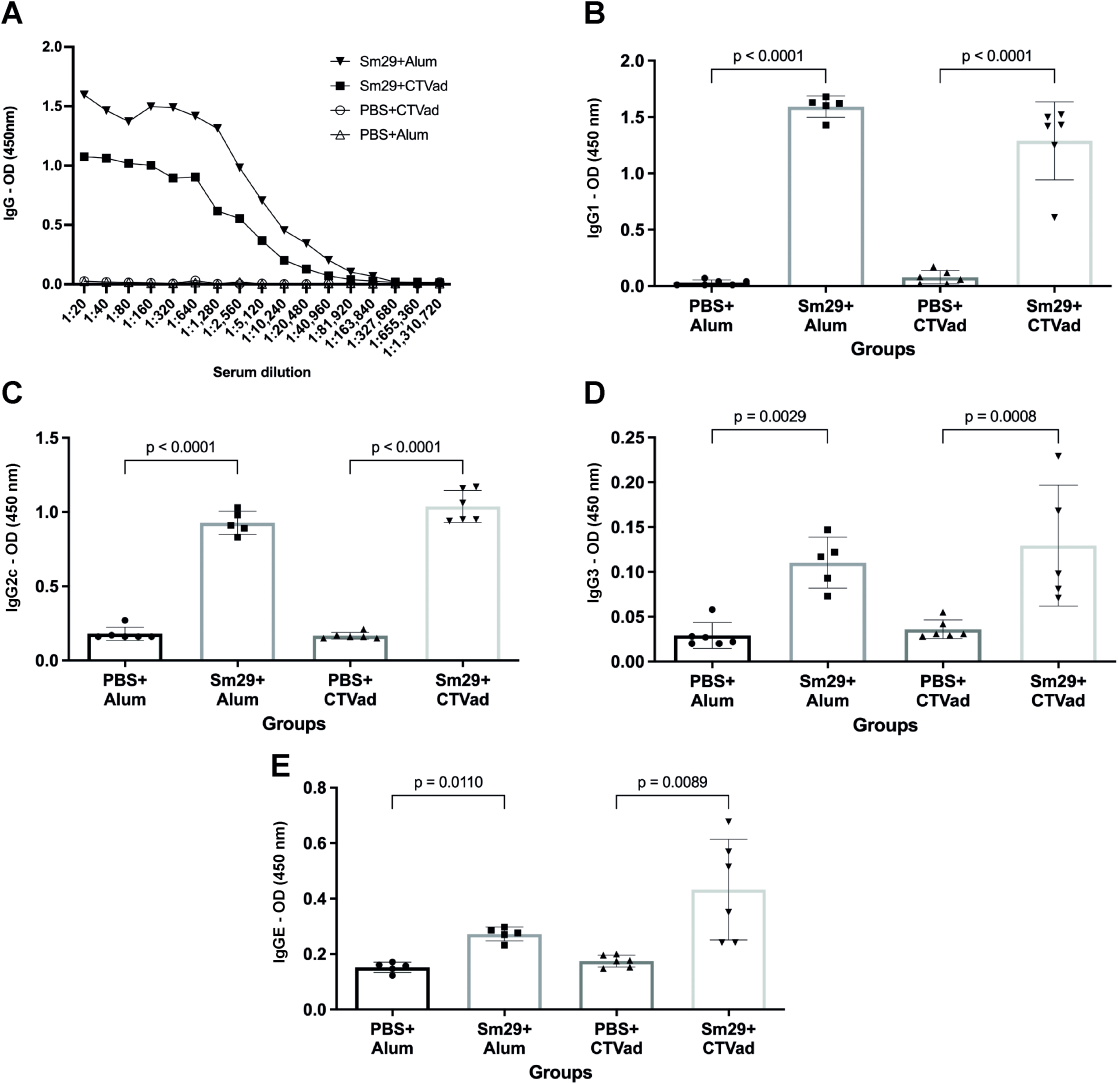
Quantification of antigen-specific antibody responses by ELISA. (A) Serum titration for total IgG levels. Serum levels of IgG1 **(B)**, IgG2c **(C)**, IgG3 (D) and IgE **(E)**. Antibody levels were measured by indirect ELISA two weeks after the final immunization. Plates were coated with recombinant Sm29 protein (10 μg/mL). Data are presented as individual values with mean ± SD. Statistical analyses were performed using ANOVA and Dunn’s multiple comparison test. *P-*values are indicated in the graph.

The humoral immune response specific to the rSm29 protein was evaluated two weeks after the last immunization. Total IgG levels were determined by titration across a serial dilutions rage (Fig 6A), whereas specific IgG1, IgG2C, IgG3, and IgE levels were evaluated at a single serum dilution (Fig 6B-6E). This analysis aimed to compare the ability of vaccine formulations containing different adjuvants (Alum or CTVad1) to induce specific antibodies to rSm29 and suggest effector functions of immunoglobulin isotypes. Both immunization regimes (Sm29 + Alum and Sm29 + CTVad1) induced higher total IgG, IgG1, IgG2c, and moderate levels of IgG3, and IgE anti-rSm29 when compared to the control groups (Fig 6A-6E).

### Cytokine production by splenocytes

To evaluate the cellular immune response profile induced by the different vaccine formulations, cytokines produced by mouse splenocytes were quantified 15 days after the last immunization, following *in vitro* stimulation with the rSm29 protein. The cytokines IFN-γ and IL-10 were quantified after 72 hours, while IL-4 and TNF were evaluated after 48 hours (Table 2). Regarding the cytokine profile, vaccination with rSm29 + Alum induced a mixed Th1/Th2 profile with significant production of IFN-γ, IL-10, and IL-4, while the rSm29 + CTVad1 immunized group showed a Th2-like pattern with production of IL-10 and IL-4. TNF was not detected in rSm29-stimulated cells, indicating that this cytokine is not relevant in the vaccine formulations tested (S2 Fig).

**Table 2:** Modulation of Sm29-Specific Cytokine Response by Different Adjuvant Formulations: Alum vs CTVad1.

Immunization Group	Cytokine	*In vitro* stimulus		
		Medium	Sm29	ConA
**PBS + Alum**	IFN-γ	25.0±18.3	0.0±0.0	2012.7±197.8 #
	IL-4	0.0±0.0	0.0±0.0	60.8±13.6 #
	IL-10	0.0±0.0	0.0±0.0	255.4±166.5 #
**PBS + CTVad1**	IFN-γ	13.2±14.1	2.7±5.6	1877.0±369.5 #
	IL-4	0.0±0.0	0.0±0.0	79.9±14.4 #
	IL-10	0.0±0.0	0.0±0.0	377.0±130.3 #
**Sm29 + Alum**	IFN-γ	15.0±14.8	620.4±698.9 *^Δ^	1776.8±262.2 #
	IL-4	0.6±1.3	30.5±28.1 *^Δ^	72.0±20.2 #
	IL-10	0.0±0.0	459.7±422.3*^Δ^	791.0±342.5 #
**Sm29 + CTVad1**	IFN-γ	14.0±4.3	1.7±4.1	1779.4±267.9 #
	IL-4	0.2±0.5	23.0±11.6 *^Δ^	65.8±28.7 #
	IL-10	0.0±0.0	391.3±346.6*^Δ^	448.8±109.6 #

Data are presented as Mean ± SD.

Statistical comparisons were performed using Kruskal-Wallis test and Dunn’s multiple comparisons test.

# denotes statistically significant differences (p < 0.05) when comparing ConA vs Medium in the same immunization group.

Δ denotes statistically significant differences (p < 0.05) when comparing Sm29 vs Medium in the same immunization group.

* denotes statistically significant differences (p < 0.05) when comparing the immunization groups Sm29 + Alum vs Alum or Sm29 + CTVad1 vs CTVad1 under Sm29 *in vitro* stimulus.

**rSm29 vaccination significantly reduces worm burden in mice**

To evaluate the protective efficacy of the rSm29 formulated with either Alum or CTVad1, mice were challenged with 100 *S. mansoni* cercariae 15 days after the last immunization and perfused 45 days post-challenge to recover adult worms. Both vaccine formulations induced significant reductions in worm burden when compared with their respective adjuvant controls, as depicted in Table 3. Immunization with Alum+Sm29 resulted in a 34.1% reduction in worm recovery relative to the Alum group, while CTVad1 + Sm29 induced a 36.9% reduction compared with the CTVad1 group*.* These findings demonstrate that both formulations containing rSm29 elicited significant and comparable levels of protection against *S. mansoni* infection in mice.

**Table 3:** Protection level induced in C57BL⁄6 mice by immunization with recombinant Sm29 vaccine.

Groups	Worm recovered (mean ± SD)	Protection (%)
PBS + Alum	43.11 ± 1.9	**–**
PBS + CTVad1	47.11 ± 2.26	**–**
Sm29 + Alum	28.4 ± 3.47	34.1*
Sm29 + CTVad1	29.75 ± 1.49	36.9*
Control	53.80 ± 1.64	**–**

Data are presented as Mean±SD. Statistical analysis was performed using Student’s *t-*test, comparisons were performed to assess the effect of the Sm29 antigen within each adjuvant group (PBS+Alum vs. Sm29 + Alum and PBS + CTVad1 vs. Sm29 + CTVad1). An asterisk denotes statistically significant differences when * p < 0.05.

### Histopathological evaluation of the liver

Histopathological evaluation was performed to investigate the impact of different vaccine formulations on the formation and organization of hepatic granulomas induced by *S. mansoni* infection. The number of concentric (well-defined) granulomas, and the number of eggs per granuloma were analyzed to correlate immune responses with the degree of tissue pathology.

**Fig 7:**
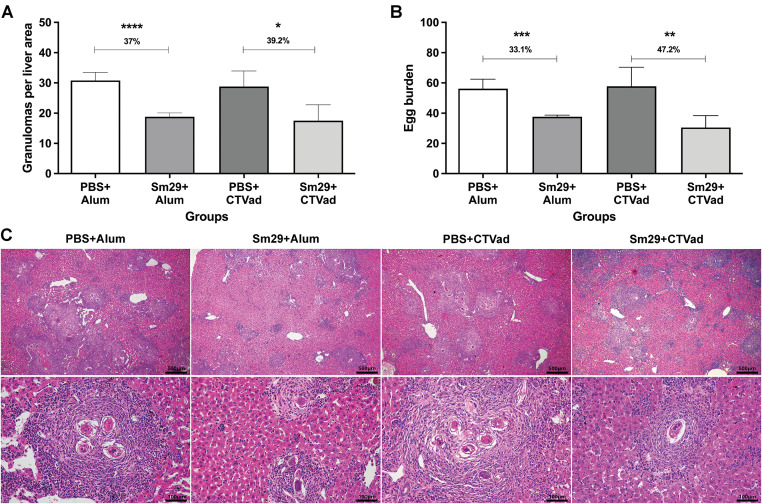
Schistosoma-induced liver pathology is influenced by protein Sm29. Pathological and molecular parameters were evaluated, after 6 weeks of infection in mice. **(A)** Number of granulomas per liver area, (B) number of eggs per gram of liver and **(C)** Representative images of granulomas detected in hematoxylin-eosin. The percentage of granuloma/liver areas were plotted in box plots (median, maximum and minimum) and analyzed via the Kruskal–Wallis test followed by Dunn’s multiple comparison test. The number of granulomas per liver area and the number of eggs per granuloma were evaluated using one-way ANOVA followed by Tukey's multiple comparison test. An asterisk denotes statistically significant differences when *p < 0.05 and ns indicates no statistical difference. Scale bars: 500µm (upper panels) and 100 µm (lower panels).

The Sm29 + Alum group exhibited a 37% reduction in the number of granulomas compared to Alum alone, and the Sm29 + CTVad1 group showed a decrease in 39.2% in granulomas number compared to CTVad1 alone (Fig 7A). Additionally, a similar response was observed when analyzing the number of eggs per granuloma. The Sm29 + Alum immunized group showed a reduction in 33.1% in egg numbers and the Sm29 + CTVad1 vaccinated group presented 47.2% reduction compared to the respective controls (Fig 7B). The groups immunized with the Sm29 protein combined with adjuvants (Alum or CTVad1) exhibited lower number of concentric granulomas and schistosome eggs (Fig 7C) compared to their respective adjuvant-only control groups, suggesting that immunization with the recombinant Sm29 protein was effective in modulating the inflammatory response and mitigating infection-associated tissue damage.

## Discussion

Schistosomiasis remains one of the most persistent neglected tropical diseases, affecting more than 151 million people worldwide and causing significant morbidity in endemic regions [51]. Despite the effectiveness of praziquantel in reducing morbidity, reinfection remains frequent, and evidence of reduced efficacy in high-transmission areas underscores the need for a long-term immunological strategy [52,53]. The development of a vaccine is therefore a critical step in achieving sustainable control of the disease. Our group has been investigating Sm29, a promising vaccine candidate that has consistently demonstrated the ability to reduce parasite burden across different experimental strategies and formulations [9,11,12,15]. To advance from experimental validation to clinical applicability, however, vaccine candidates must be produced under GLP conditions to ensure reproducibility, traceability, and compliance with regulatory standards. In this context, the production of rSm29 under GLP standards represents a fundamental advance in the translational development of vaccine candidates against *S. mansoni*. By establishing a standardized workflow, the present study demonstrates the feasibility of producing rSm29 under conditions aligned with the quality frameworks adopted by the regulatory agencies for preclinical vaccine production.

Our study was conducted at CT Vacinas (Federal University of Minas Gerais), a facility operating under a Good Laboratory Practice (GLP) quality system certified by Inmetro, the Brazilian National Institute of Metrology, Quality and Technology. Consequently, all analytical procedures, equipment qualification, reagent traceability, documentation practices, and data management activities were performed according to established quality assurance requirements. Although GLP certification does not replace scientific validation of individual methods, it provides an additional level of confidence regarding the reliability, reproducibility, traceability, and integrity of the data generated throughout the study.

Previous studies on Sm29 have primarily focused on its immunogenicity and protective efficacy in experimental models. In contrast, the present study addresses an important translational aspect by establishing a standardized downstream purification and quality-control strategy for recombinant Sm29 production under Good Laboratory Practice (GLP) conditions. In addition to achieving a highly purified recombinant antigen, the process included the evaluation of critical product quality attributes, such as residual endotoxin, host-cell proteins, residual host-cell DNA, purity, and process consistency. These parameters are considered essential for the development of recombinant vaccine antigens intended for preclinical and clinical studies, as they ensure product safety, reproducibility, and compliance with current regulatory expectations for biological products [18,23,54]. Therefore, this work extends previous Sm29 studies by providing a robust manufacturing and analytical framework that supports the future translational development of this vaccine candidate.

From a structural and analytical perspective, rSm29 maintained its integrity, with no evidence of degradation or aggregation, as confirmed by microfluidic electrophoresis. Preservation of conformational epitopes is essential for sustaining antigenicity and reproducibility across batches [55]. Moreover, the tag-free construct design minimizes the presence of non-native epitopes and aligns the antigen with regulatory expectations for human-use biopharmaceuticals [56]. The incorporation of a solubility-enhancing DDD motif represents a rational protein-engineering strategy designed to improve protein solubility and facilitate downstream processing. Similar approaches employing short acidic or polyionic tags have been shown enhance protein solubility while preserving the immunogenic properties observed in the present study.[57]. Although the recombinant Sm29-DDD protein exhibited satisfactory expression, purification, antigenicity, and analytical quality attributes, the present study was not designed to independently evaluate the specific contribution of the DDD motif to these outcomes. Therefore, any direct effect of this modification on protein expression, solubility, purification efficiency, or refolding cannot be conclusively established from the current data. The use of short acidic peptide extensions has previously been explored as a strategy to modify physicochemical properties of recombinant proteins, although their effects remain protein-dependent and require empirical evaluation [58,59]. The results of the present study demonstrate that the incorporation of the C-terminal DDD motif into recombinant Sm29 enabled the production of a soluble and immunoreactive antigen while preserving its protective potential. Immunization with Sm29-DDD resulted in a 37% reduction in worm burden, indicating that the structural modification did not impair the biological activity of the antigen. Although this level of protection is lower than the highest reductions reported for some leading schistosomiasis vaccine candidates, including Sm14, Sm-p80 and Sm-TSP-2 under optimized experimental conditions, it falls within the range of partial protection considered biologically relevant for anti-schistosome vaccines, where even moderate reductions in parasite burden may substantially decrease egg deposition, disease morbidity, and transmission [11,60,61]. Importantly, protection should be interpreted together with the technological advances achieved in antigen production. One important result of this study was the high recombinant protein yield, reaching approximately 100 mg/L of bacterial culture. This productivity compares favorably with many recombinant schistosome antigens, whose production is frequently limited by poor solubility, aggregation, or low recovery during downstream processing. Similar to the development of the Sm14/GLA-SE vaccine, where scalable manufacturing and product quality were considered essential for clinical translation [62,63], the present production strategy combined high yield with analytical quality control under GLP conditions, providing a robust platform for preclinical vaccine development. Furthermore, studies on Sm-TSP-2 have highlighted that preserving antigen conformation and immunoreactivity is critical for eliciting functional immune responses and antibody recognition [61,64]. In agreement with these observations, the ELISA results obtained in this study demonstrated that the addition of the DDD motif did not compromise antigen recognition, supporting the preservation of relevant immunogenic epitopes. Collectively, these findings suggest that the Sm29-DDD construct not only maintains the biological characteristics required for vaccine efficacy but also exhibits manufacturing attributes that are highly desirable for future scale-up and translational development.

Many parameters are critical since process-related impurities can compromise antigen stability or induce unwanted immune responses, particularly in *E. coli*-derived products where endotoxin contamination may provoke strong inflammatory reactions that confound immunogenicity studies [65]. The very low endotoxin levels reported here ensure that subsequent immune responses in preclinical models are attributable to the rSm29 antigen itself and not to bacterial components. The use of *E. coli* as an expression platform represents an important advantage for the development of recombinant vaccines because this system combines rapid growth, low production costs, straightforward genetic manipulation, and the potential for large-scale manufacturing, characteristics that have supported the production of several experimental and licensed recombinant biopharmaceuticals [44,66]. However, the expression of cysteine-rich proteins from helminths remains challenging in conventional *E. coli* strains due to the reducing environment of the bacterial cytoplasm, which often results in protein misfolding and aggregation. Sm29 contains twenty cysteine residues that are predicted to form multiple disulfide bonds, making the selection of an appropriate expression host particularly relevant. In this context, the SHuffle strain offers significant advantages because it carries mutations in the thioredoxin reductase (*trxB*) and glutathione reductase (*gor*) genes, creating a more oxidizing cytoplasmic environment that favors disulfide bond formation. In addition, SHuffle constitutively expresses the disulfide bond isomerase DsbC in the cytoplasm, promoting the rearrangement of incorrectly formed disulfide bonds and improving the folding of complex recombinant proteins [29]. The successful expression of immunoreactive Sm29-antigens, including Sm14, Sm-TSP-2, Sm-p80, and Sm29 in *E. coli* SHuffle and the high recovery obtained after purification suggest that this strain provided a suitable platform for the production of this cysteine-rich schistosome antigen. Therefore, the combination of the SHuffle expression system with the DDD solubility-enhancing strategy constitutes an attractive approach for the production of structurally complex vaccine antigens intended for preclinical and translational development. The ability to produce approximately 100 mg/L of a cysteine-rich antigen such as Sm29 further highlights the suitability of the SHuffle platform for manufacturing recombinant schistosome vaccine candidates.

The rSm29 protein exhibited limited binding strength to the anion-exchange matrix under the selected pH and conductivity conditions. Preliminary optimization experiments demonstrated that even low salt concentrations in the wash buffer promoted partial desorption of the target protein. Therefore, omission of NaCl from the washing step was necessary to prevent premature elution and maintain acceptable recovery yields. Chromatographic washing conditions should preserve target protein binding while maximizing impurity removal. Excessively stringent wash conditions may compromise product recovery by inducing premature desorption of weakly bound proteins. Importantly, the dialysis strategy resulted in the successful recovery of purified protein with satisfactory analytical characteristics, including purity, residual DNA content, endotoxin levels, and preservation of antigenicity, demonstrating its suitability for the current stage of process development. In the present study, dialysis was intentionally selected because it simultaneously enabled the gradual removal of urea, buffer exchange, conductivity adjustment between chromatographic steps, and protein refolding. This approach is frequently reported for proteins recovered from inclusion bodies, particularly cysteine-rich proteins requiring controlled structural recovery. Following purification, the recombinant Sm29 protein was subjected to a controlled refolding procedure through gradual removal of urea by dialysis and buffer exchange [67–69]. This step was intended to promote the recovery of the protein's tertiary structure and facilitate the correct formation of disulfide bonds before formulation. It should also be considered that vaccine-induced immune responses may target both conformational and linear epitopes. Therefore, preservation of the complete native structure is not necessarily required for induction of protective immunity.

From a structural and analytical perspective, rSm29 maintained its integrity throughout the purification process. Identity was confirmed at the peptide level by LC-MS/MS with 100% sequence coverage using a dual-enzyme strategy (trypsin and chymotrypsin), representing a higher-confidence approach than immunoblotting alone and consistent with ICH Q6B specifications for recombinant biopharmaceutical characterization [18]. Importantly, the C-terminal DDD motif was confirmed present in the final purified product through identification of the peptide *LYIDDQISCCSTDLDDD* by both proteolytic strategies (XCorr 5.84 and 8.35, respectively), providing direct experimental evidence that the solubility-enhancing engineering was preserved throughout expression and purification. Furthermore, far-UV circular shuffle analysis revealed a spectral profile consistent with the three-finger fold of the Ly6/uPAR protein family, confirming that refolding from inclusion bodies yielded a structurally competent antigen [48,50]. Together, these orthogonal analytical approaches — microfluidic electrophoresis, LC-MS/MS peptide mapping, and circular dichroism — provide converging evidence of protein identity, sequence integrity, and structural competence, collectively supporting the preservation of conformational epitopes relevant to vaccine-induced immunity.

The sequential purification strategy proved particularly important for endotoxin removal. Following the second chromatographic step, residual endotoxin levels remained at 58 EU/mg protein, demonstrating that additional purification was necessary. Consequently, a third QXL chromatography step was incorporated as a dedicated polishing stage. This additional purification substantially reduced endotoxin levels while preserving protein recovery and purity, highlighting the importance of impurity-specific downstream strategies for recombinant proteins produced in *Escherichia coli* [70,71]. Since endotoxins are potent activators of innate immune responses, their efficient removal is essential to ensure that subsequent immunological evaluations reflect the biological properties of the recombinant antigen rather than contamination-derived effects [70].

The rSm29 antigen produced herein exhibited high purity and integrity, with residual DNA and endotoxin levels well below internationally accepted limits (3.5 ng per dose and 0.154 EU/μg protein, respectively), while host cell protein was detected at approximately 0.014% (w/w), a value close to the recommended threshold. These results are consistent with the specifications for recombinant biological products (<10 ng DNA/dose, < 3.5 EU/μg protein, and host cell protein around 0.01%), supporting the robustness of the purification process [17–25].

An important consideration in the present study is the residual host-cell protein (HCP) content detected in the final Sm29 preparation. Although the purification strategy substantially reduced process-related impurities, the final HCP level remained slightly above the commonly cited industry benchmark of 100 ng/mg. Residual HCPs are considered critical quality attributes because they may affect product stability, biological activity, and immunogenicity [72,73]. However, current regulatory guidance recognizes that acceptable HCP levels cannot be universally defined and should instead be interpreted within the context of product-specific risk assessment, taking into account the identity of the residual proteins, route of administration, patient population, and overall product safety profile [54,74]. The persistence of residual HCPs after purification is not unexpected. Certain HCP species are known to be particularly difficult to remove because they may exhibit physicochemical properties similar to those of the target protein, including comparable molecular weights, isoelectric points, and chromatographic behavior. In addition, some HCPs can remain associated with the recombinant protein through stable protein-protein interactions, resulting in co-purification during multiple downstream processing steps [72,75]. In the present study, all purification stages were based on anion-exchange chromatography. Although this approach proved effective in improving product purity and reducing process-related contaminants, repeated application of the same chromatographic principle may not completely eliminate specific populations of co-purifying HCPs. Additional impurity clearance could potentially be achieved through the incorporation of orthogonal purification strategies, such as size-exclusion chromatography or hydrophobic interaction chromatography, which exploit differences in molecular size and hydrophobicity between the target protein and residual host-cell proteins [76].

Further optimization of chromatographic washing conditions may also improve HCP clearance. However, process development studies demonstrated that recombinant Sm29 began to elute at very low ionic strengths, and therefore more stringent washing conditions resulted in significant product loss. Future studies may evaluate alternative washing strategies, including modified salt gradients or additives capable of disrupting protein-HCP interactions while preserving product recovery. Additionally, characterization of residual HCPs by LC-MS/MS would provide valuable information regarding the identity and potential risk associated with the remaining impurities. While ELISA-based assays provide an estimate of total HCP burden, they do not identify individual protein species. Proteomic characterization could therefore distinguish between low-risk residual proteins and HCPs with greater potential impact on product quality, stability, or immunogenicity [74,75]. Importantly, HCP quantification should not be interpreted as a direct measure of recombinant protein purity but rather as an assessment of residual process-related impurities originating from the host expression system. Current regulatory principles emphasize that purification strategies should be scientifically justified and demonstrate adequate impurity clearance and product quality, without mandating a specific number or orthogonality of chromatographic steps [18,54]. Therefore, although additional process optimization remains possible, the HCP level observed in the present study should be interpreted within the broader context of product characterization and process development.

The present study reiterates the potential of rSm29 as a vaccine candidate against schistosomiasis, a finding that consistently validates research in the area. In this study, we demonstrate that both formulations, Sm29 + Alum and Sm29 + CTVad1, are effective in inducing total IgG, IgG1, IgG2c, IgG3 and IgE anti-Sm29 responses. Similar results, showing that Sm29 associated with Alum induces a strong total IgG response, were found in the study by Cardoso et al.[11]and Alves et al. [15]. Sm29 is expressed on the tegument of newly transformed skin-stage schistosomula, as well as on lung-stage parasites and adult worms, placing this antigen in direct contact with host immune effectors. In this context, elevated levels of IgG isotypes have been associated with schistosomula death through antibody-dependent cell-mediated cytotoxicity and the activation of complement [77,78]. Antibody production is also involved in long-term protective immunity and related to resistance to schistosomiasis reinfection [79–81]. Herein, animals immunized with both adjuvants plus rSm29 showed a robust production of specific IgG1 and IgG2c, and moderate IgG3 and IgE levels. In humans, a similar pattern of antibody production is observed in individual naturally resistant to *S. mansoni* reinfection from endemic areas, who display high levels of anti-Sm29 IgG1 and IgG3, as well as an increased production of INF-gamma by peripheral blood mononuclear cells [9]. This correlation indicates that the robust induction of specific antibodies observed in our animal model aligns with the human immune profile naturally triggered by contact with the parasite, demonstrating that sporadic and low-dose exposure in endemic regions is capable of triggering and maintaining a functional immunological memory against this antigen.

Regarding the cytokine profile, supernatants of cultured splenocytes extracted from immunized mice were stimulated with rSm29. Our cytokine measurements demonstrated that immunization with rSm29 + Alum induced a mixed Th1 and Th2 response profile, as previously observed by Alves et al.[12]. While Sm29 + CTVad1 vaccination elicited IL-4 and IL-10, a Th2-like response. Notably, a comparable dual polarization was confirmed in the recent Phase II Sm14 + GLA-SE clinical trial conducted in West Africa, where vaccinated adults and schoolchildren developed concurrent Th1-associated (IFN-γ, TNF-α) and Th2-associated (IL-4, IL-5) cytokine responses [63]. This parallel reinforces those balanced Th1/Th2 responses, such as those induced by rSm29 in our experimental model, is consistent with protective profile observed in successful human schistosomiasis vaccine trial. We acknowledge that additional studies are required to further elucidate the mechanisms associated with protection induced by recombinant Sm29. Future investigations will include more comprehensive immune profiling strategies, such as multiparametric flow cytometry to characterize antigen-specific CD4+ and CD8 + T-cell responses, memory T-cell populations (CD45RO and CD45RA), multifunctional cytokine-producing cells, and the durability of vaccine-induced immunity. Nevertheless, both adjuvants plus rSm29 induced protection against schistosome infection. An important readout of vaccination trials against schistosomes is the decrease in the parasite burden associated with the hepatic lesions. In our study, rSm29 immunization plus Alum or CTVad1 resulted in a 34.1% or 36.9% reduction in adult worm burden, respectively. Regarding liver pathology, rSm29 vaccination plus Alum or CTVad1 groups showed 33.1% and 47.2% reduction in eggs trapped in the liver, respectively. These data are consistent with previous findings, which showed a 50% reduction of eggs in the liver and 60% in the intestine. Furthermore, parasites recovered from animals immunized with rSm29 showed global down-regulation of essential parasites genes, such as *SmINSIG*, which modulates the HMG-CoA reductase pathway required for egg production. Consequently, this molecular stress may impair female worm fecundity or parasite survival, thereby reducing tissue egg deposition [11]. The granuloma area around the eggs was also reduced, 37% upon rSm29 + Alum vaccination and 39.2% following rSm29 + CTVad1 immunization, confirming the protein's potential to mitigate tissue damage. These moderate protection levels closely parallel leading vaccine candidates in clinical trials. For instance, rSm-TSP-2 formulated with alum-based adjuvants typically yields a 25–28% worm reduction and 27–56 tissue egg reduction in mice [82,83]. Regarding the adjuvant CTVad1, it is an oil-in-water nanoemulsion based on squalene, developed by the partner group (CTVacinas) as an analogue of the established commercial adjuvant MF59. A comprehensive physicochemical comparability study was previously conducted, confirming that CTVad1 closely matches the critical quality attributes (CQAs) of MF59, including droplet size distribution, polydispersity index, zeta potential, and squalene stability profiles [16]. Furthermore, the selection of CTVad1 is aligned with a clinical translation strategy. CTVad1 has already undergone rigorous preclinical safety and immunogenicity testing and has successfully progressed to human clinical trials. It serves as an adjuvant platform for SpiN-Tec (a protein-based chimeric COVID-19 vaccine developed by CTVacinas), which has demonstrated an excellent safety and immunogenicity profile in Phase I/II clinical trials and is currently transitioning to a Phase III clinical study [84].

In conclusion, this study successfully produced and purified rSm29 protein under controlled, GLP-grade conditions, ensuring high purity and minimal process-related impurities, in line with international regulatory standards. Immunization of mice with rSm29 combined with different adjuvants confirmed its immunogenic potential, conferring partial protection against infection and reduced liver pathology. These findings reinforce rSm29 as a promising vaccine candidate against *S. mansoni* and demonstrate that formulation with safe, human-compatible adjuvants such as Alum or CTVad1 can induce protective immunity while maintaining antigen quality suitable for preclinical development.

## Supporting information

S1 Fig *In silico* physicochemical characterization of the rSm29 recombinant protein. (A) Sequence map of the 141-amino acid rSm29 construct, with each residue color-coded according to its physicochemical class: red, cysteine (C); blue, hydrophobic (A, V, I, L, M, F, Y, W, P); teal, polar uncharged (S, T, N, Q); orange, positively charged (K, R, H); purple, negatively charged (D, E); grey, Gly/Pro. Cysteine positions are indicated by downward arrowheads (v); the engineered C-terminal DDD motif is highlighted. (B) Amino acid composition of rSm29, displayed as a horizontal bar plot ordered alphabetically (A–Y), with residues colored by physicochemical class as described in (A). (C) Hydrophobicity profile calculated using the Kyte–Doolittle scale with a sliding window of 9 residues. Positive values (blue) indicate hydrophobic regions; negative values (purple) indicate hydrophilic regions. Vertical red lines indicate cysteine residue positions; cysteine labels are displayed on the upper x-axis. The overall GRAVY index of −0.119 reflects the predominantly hydrophilic nature of the protein surface.
(PDF)

S2 Fig TNF levels were measured by ELISA in the supernatant of splenocytes incubated with medium only as a negative control or stimulated with rSm29 or the positive control LPS. Data are presented as mean ± SD. Statistical comparisons were performed using Kruskal-Wallis test and Dunn´s multiple comparisons test. # denotes statistically significant differences (p < 0.05) when comparing LPS vs Medium in the same immunization group.
(PDF)

S1 Raw Gels (A) Raw gel for Fig 2A. (B) Raw gel for Fig 2B. (C) Raw gel for Fig 2C. (D) Raw gel for Fig 2D. (E) Raw gel for Fig 2E. (F) Raw gel for Fig 2F. (G) Raw gel for Fig 2G. (H) Raw gel for Fig 2H. (I) Raw gel for Fig 2I. (J) Raw gel for Fig 3A. (PDF)
