# Supplementary material for: Optimization and production of a GLP-grade recombinant Sm29 vaccine against schistosomiasis: From expression to preclinical assessment
Source: PLoS Negl Trop Dis. 2026 Aug 7;20(8):e0014617. doi: 10.1371/journal.pntd.0014617 (PMC13472504; doi:10.1371/journal.pntd.0014617)
Supplement: Supplementary file 1 [file pntd.0014617.s001.pdf]

S1 Figure

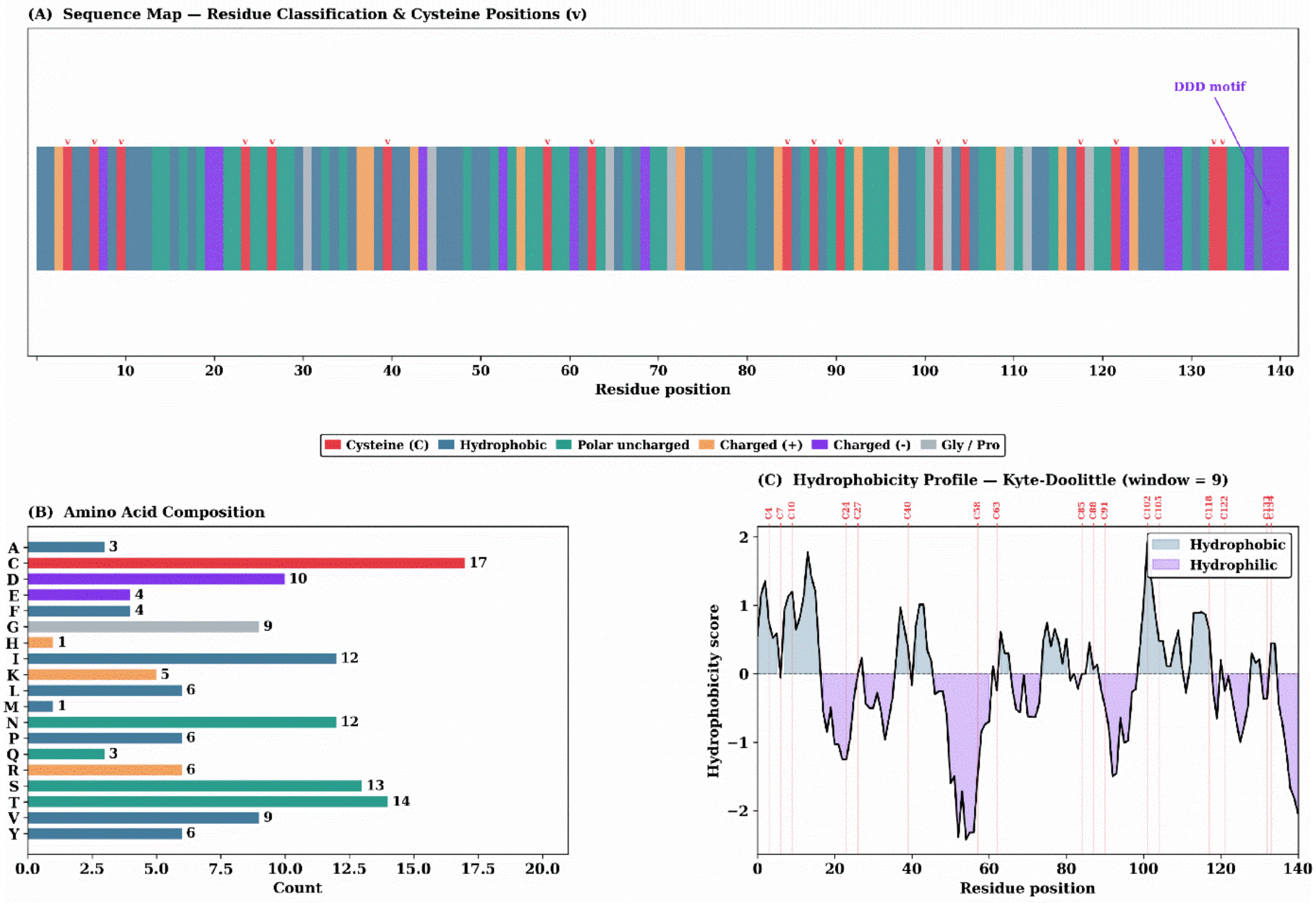

(A) Sequence Map — Residue Classification & Cysteine Positions (v)
DDD motif
Residue position
Cysteine (C)
Hydrophobic
Polar uncharged
Charged (+)
Charged (-)
Gly / Pro
(B) Amino Acid Composition
Count
(C) Hydrophobicity Profile — Kyte-Doolittle (window = 9)
Hydrophobic
Hydrophilic
Hydrophobicity score
Residue position
