# Supplementary material for: Optimization and production of a GLP-grade recombinant Sm29 vaccine against schistosomiasis: From expression to preclinical assessment
Source: PLoS Negl Trop Dis. 2026 Aug 7;20(8):e0014617. doi: 10.1371/journal.pntd.0014617 (PMC13472504; doi:10.1371/journal.pntd.0014617)
Supplement: Supplementary file 2 [file pntd.0014617.s002.pdf]

S2 Figure

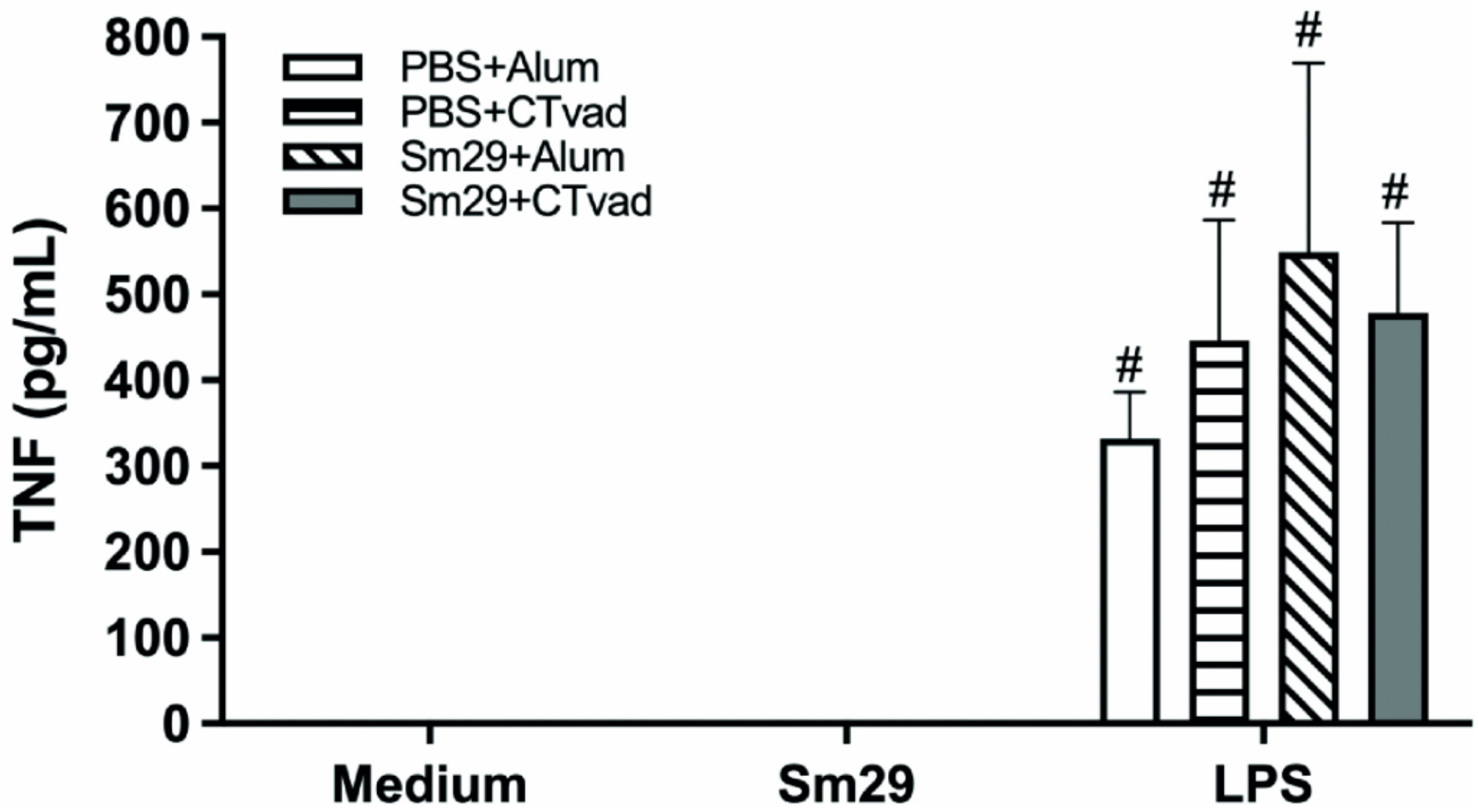

800
700
600
500
400
300
200
100
0
TNF (pg/mL)
PBS+Alum
PBS+CTvad
Sm29+Alum
Sm29+CTvad
#
#
#
#
Medium
Sm29
LPS
