# Supplementary material for: Optimization and production of a GLP-grade recombinant Sm29 vaccine against schistosomiasis: From expression to preclinical assessment
Source: PLoS Negl Trop Dis. 2026 Aug 7;20(8):e0014617. doi: 10.1371/journal.pntd.0014617 (PMC13472504; doi:10.1371/journal.pntd.0014617)

S1 Raw images

A. Raw image for Figure 2A. Image captured using a Bio-Rad Gel Documentation System

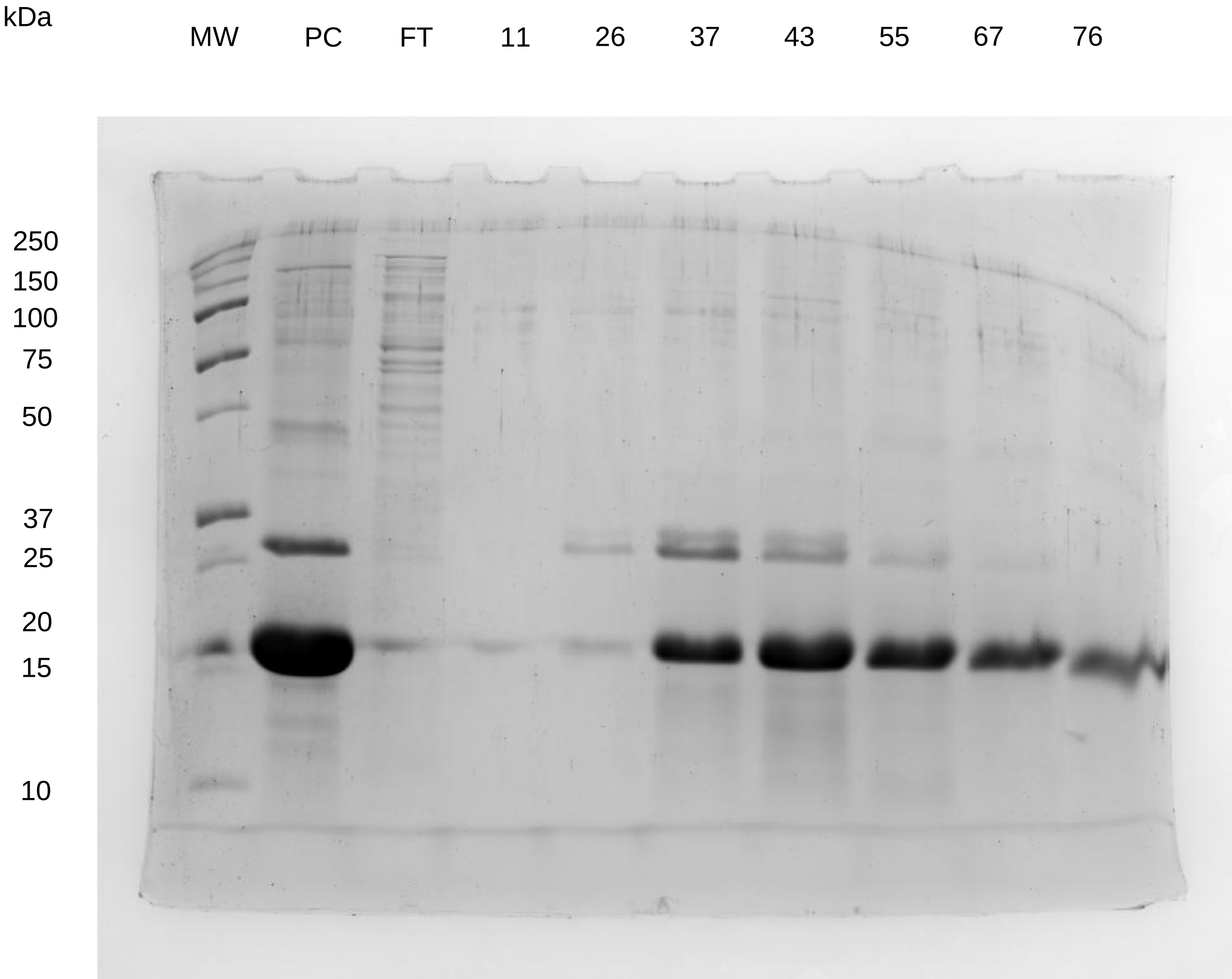

B. Raw image for Figure 2B. Image captured using a Bio-Rad Gel Documentation System

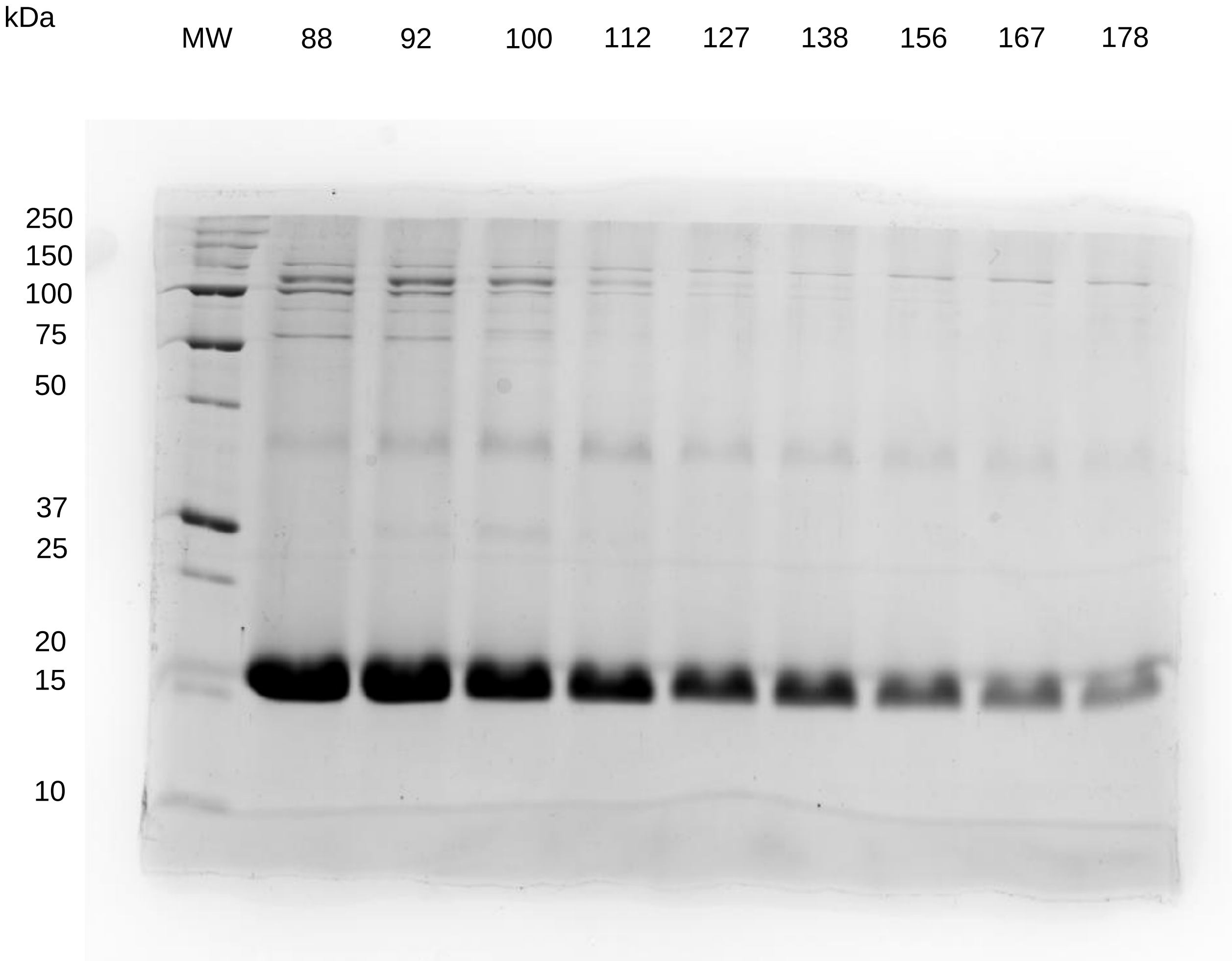

C. Raw image for Figure 2C. Image captured using a Bio-Rad Gel Documentation System

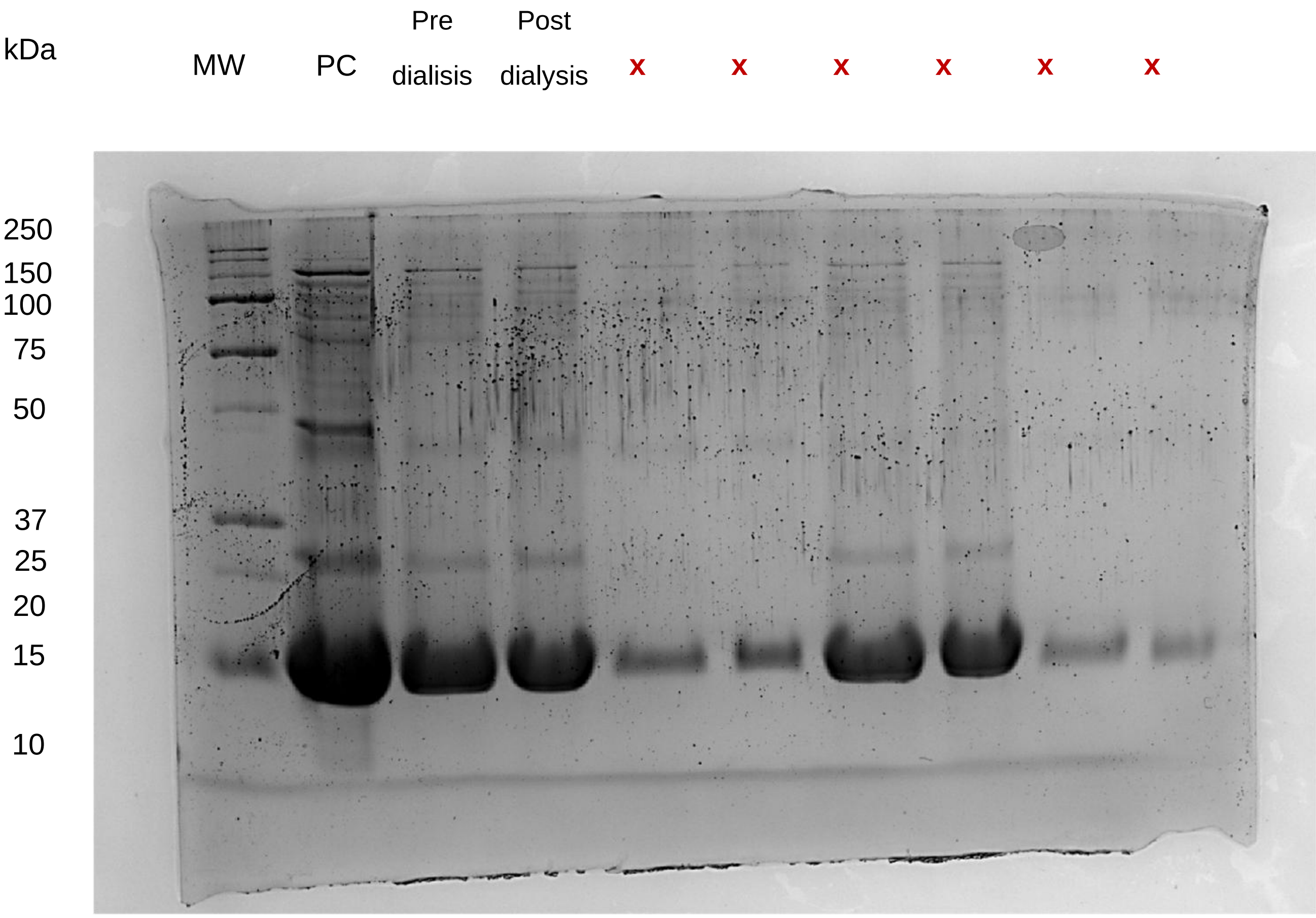

D.Raw image for Figure 2D. Image captured using a Bio-Rad Gel Documentation System

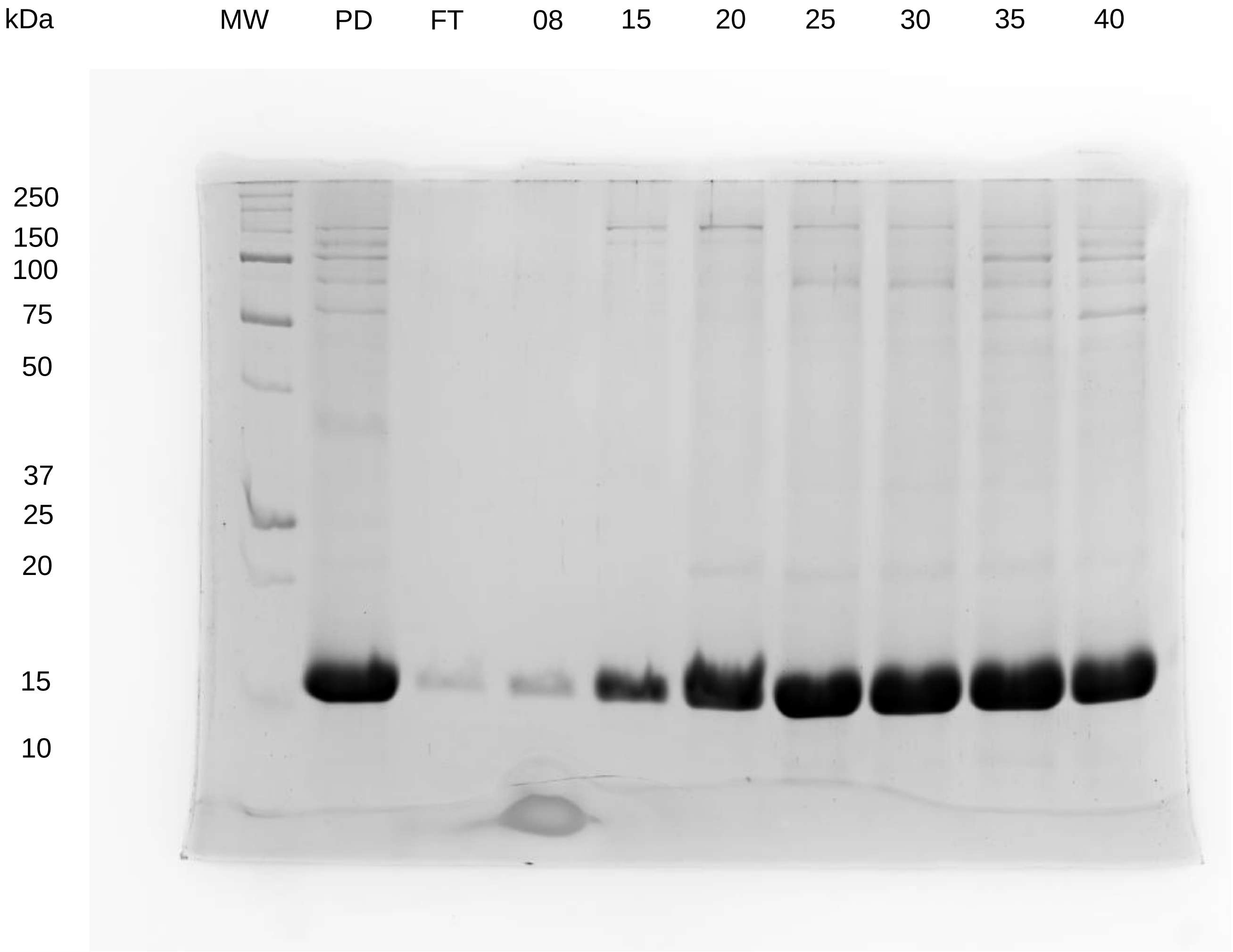

E.Raw image for Figure 2E. Image captured using a Bio-Rad Gel Documentation System

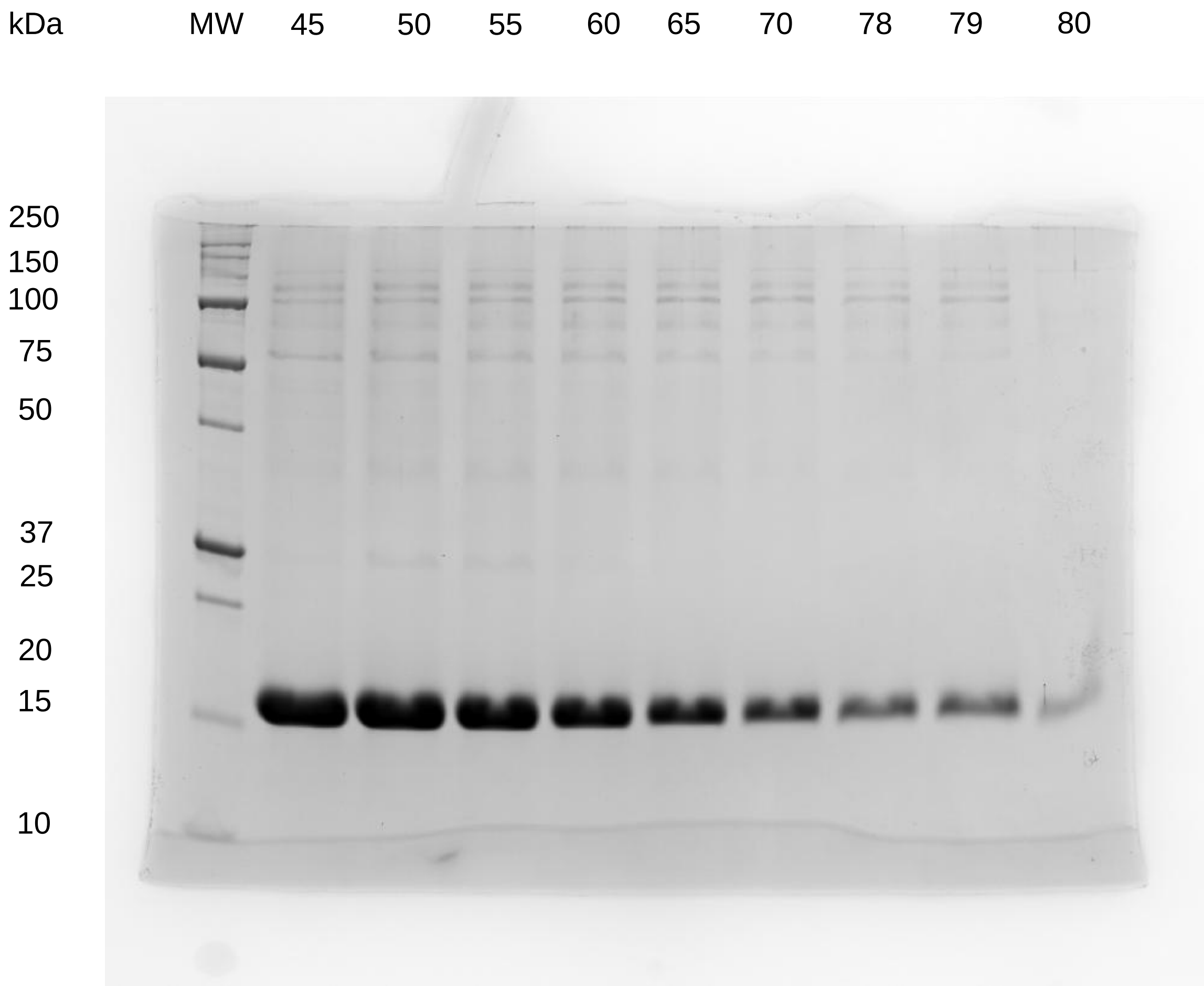

F. Raw images for Figure 2F. Image captured using a Bio-Rad Gel Documentation System

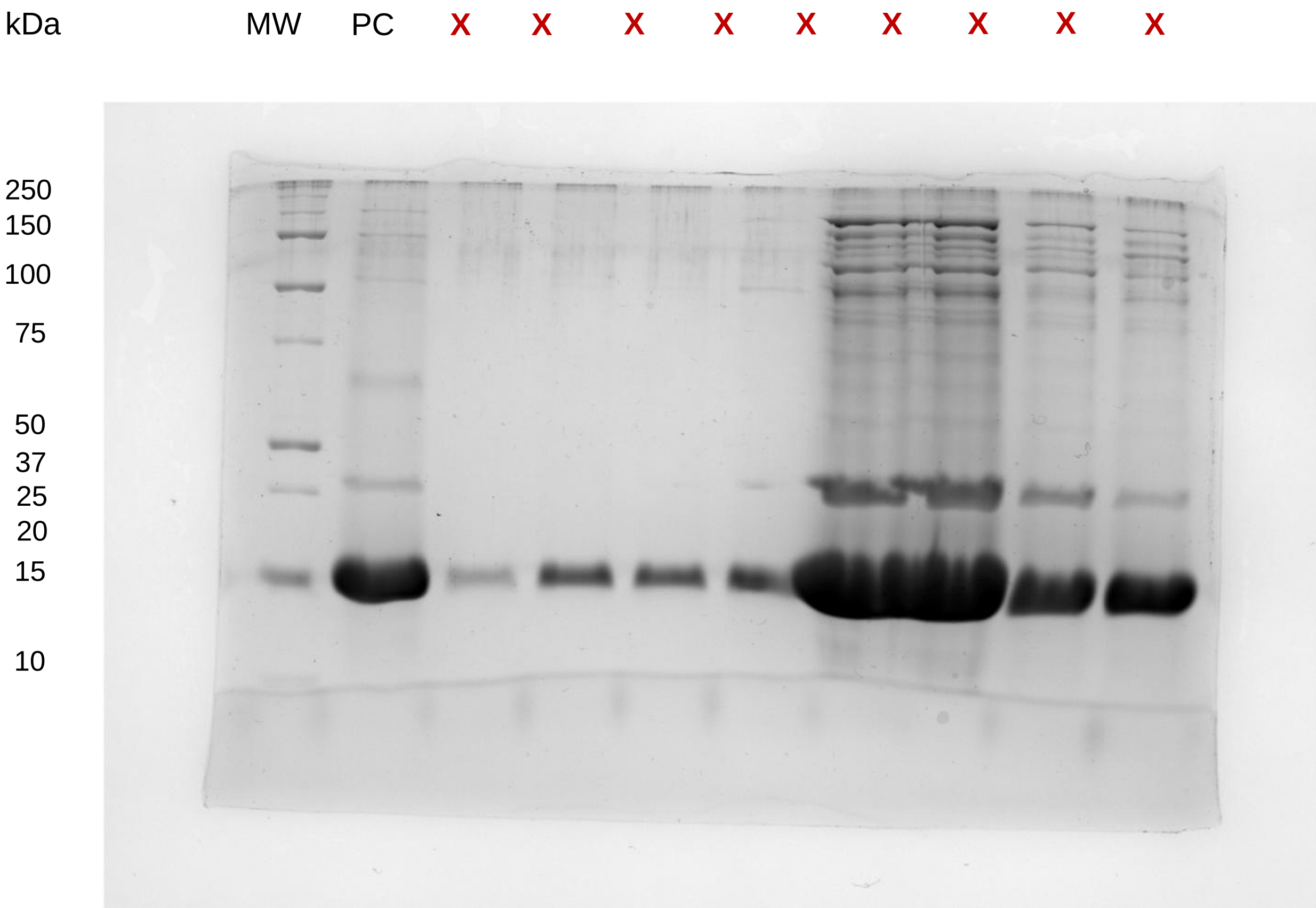

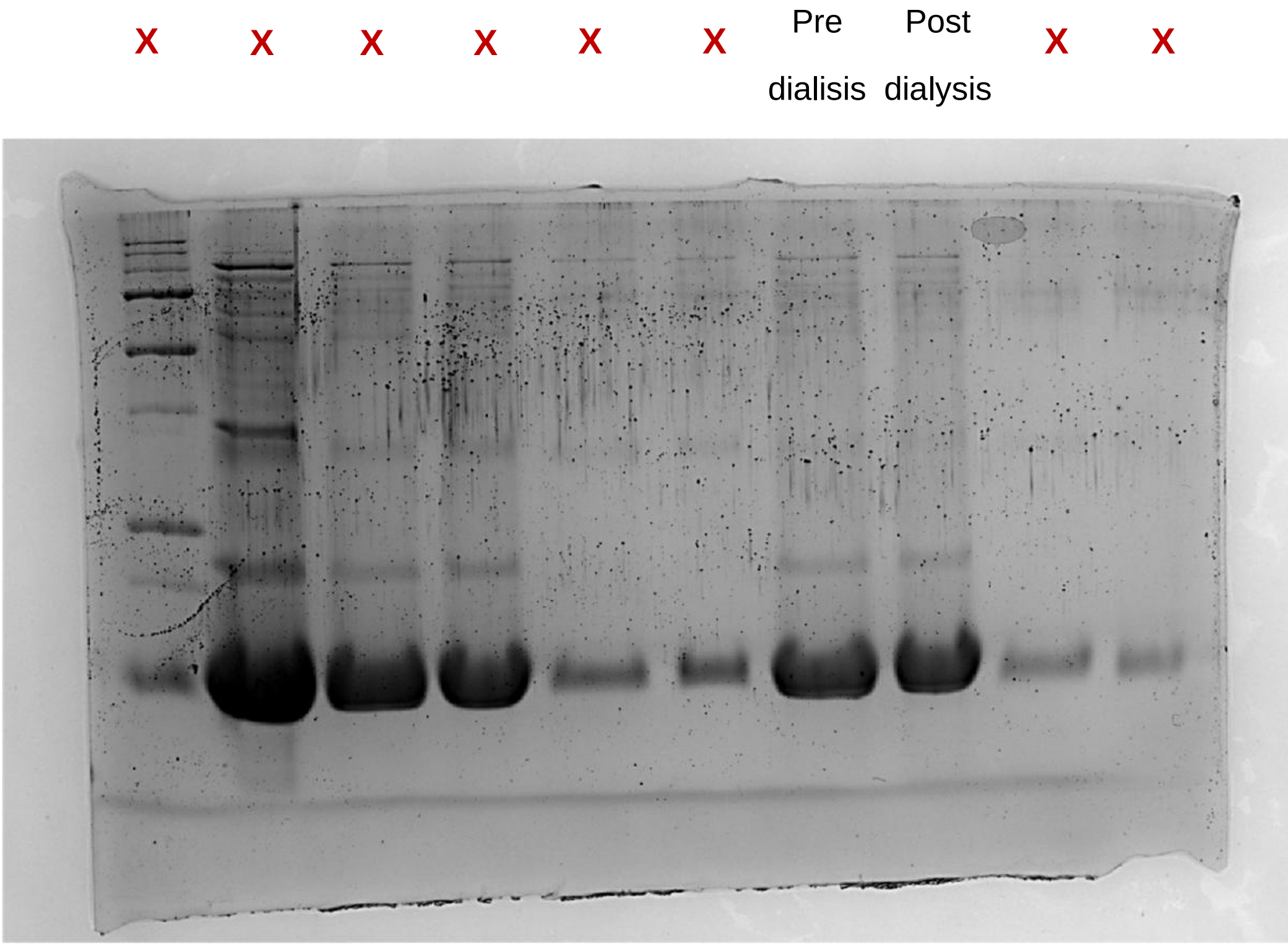
X
X
X
X
X
X
Pre dialisis
Post dialysis
X
X

G. Raw image for Figure 2G. Image captured using a Bio-Rad Gel Documentation System

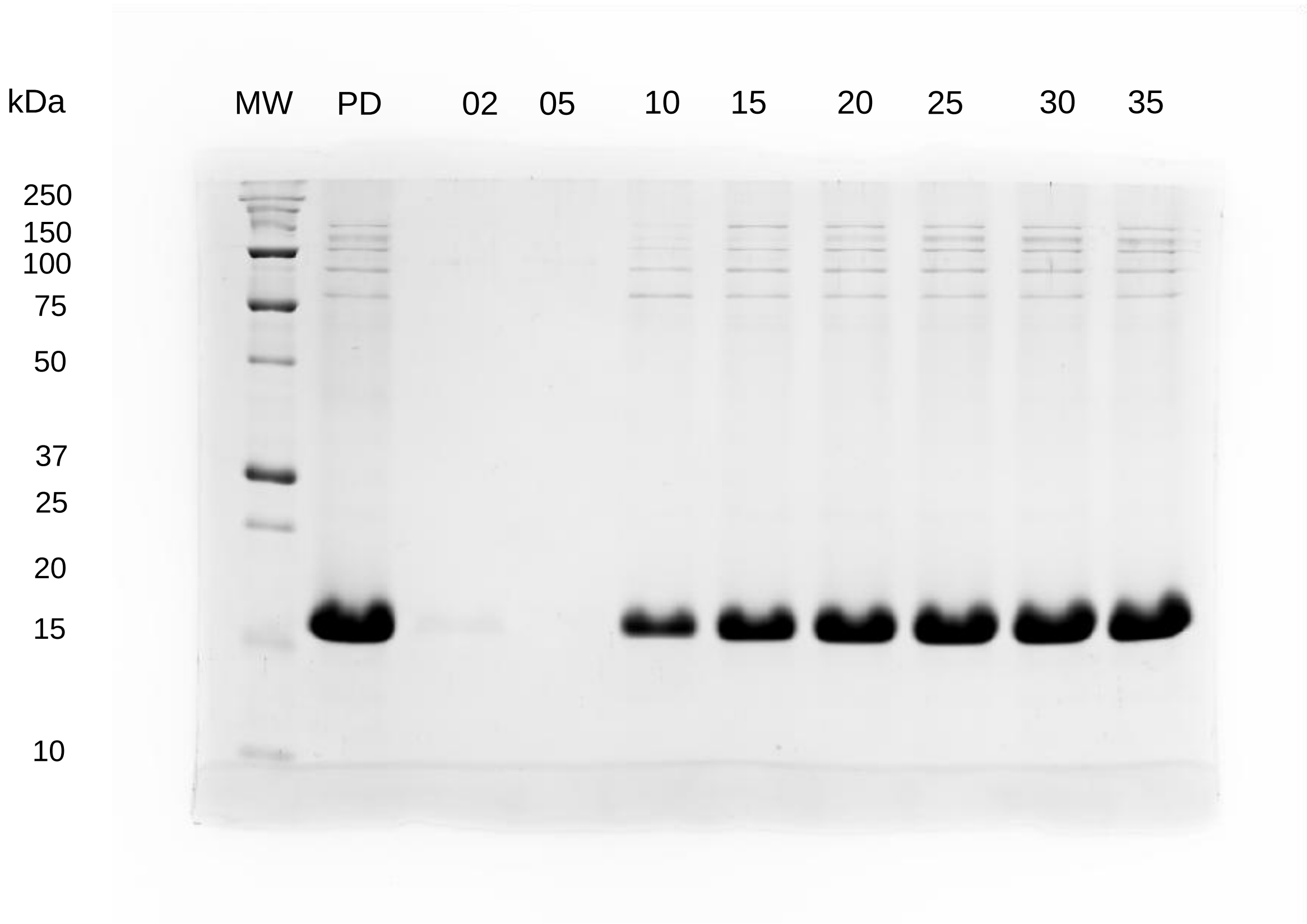

H. Raw image for Figure 2H. Image captured using a Bio-Rad Gel Documentation System

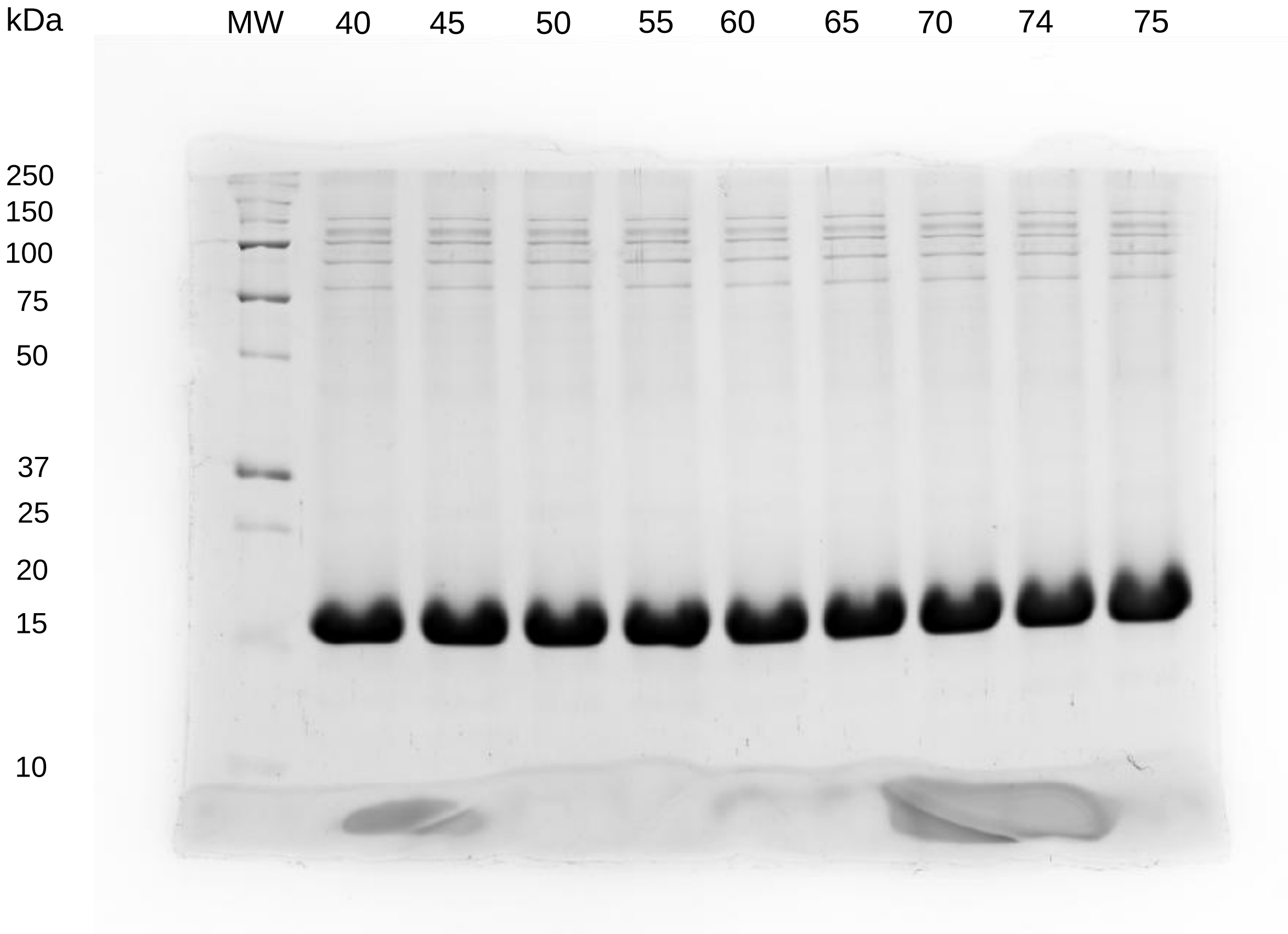

I. Raw images for Figure 2I. Image captured using a Bio-Rad Gel Documentation System

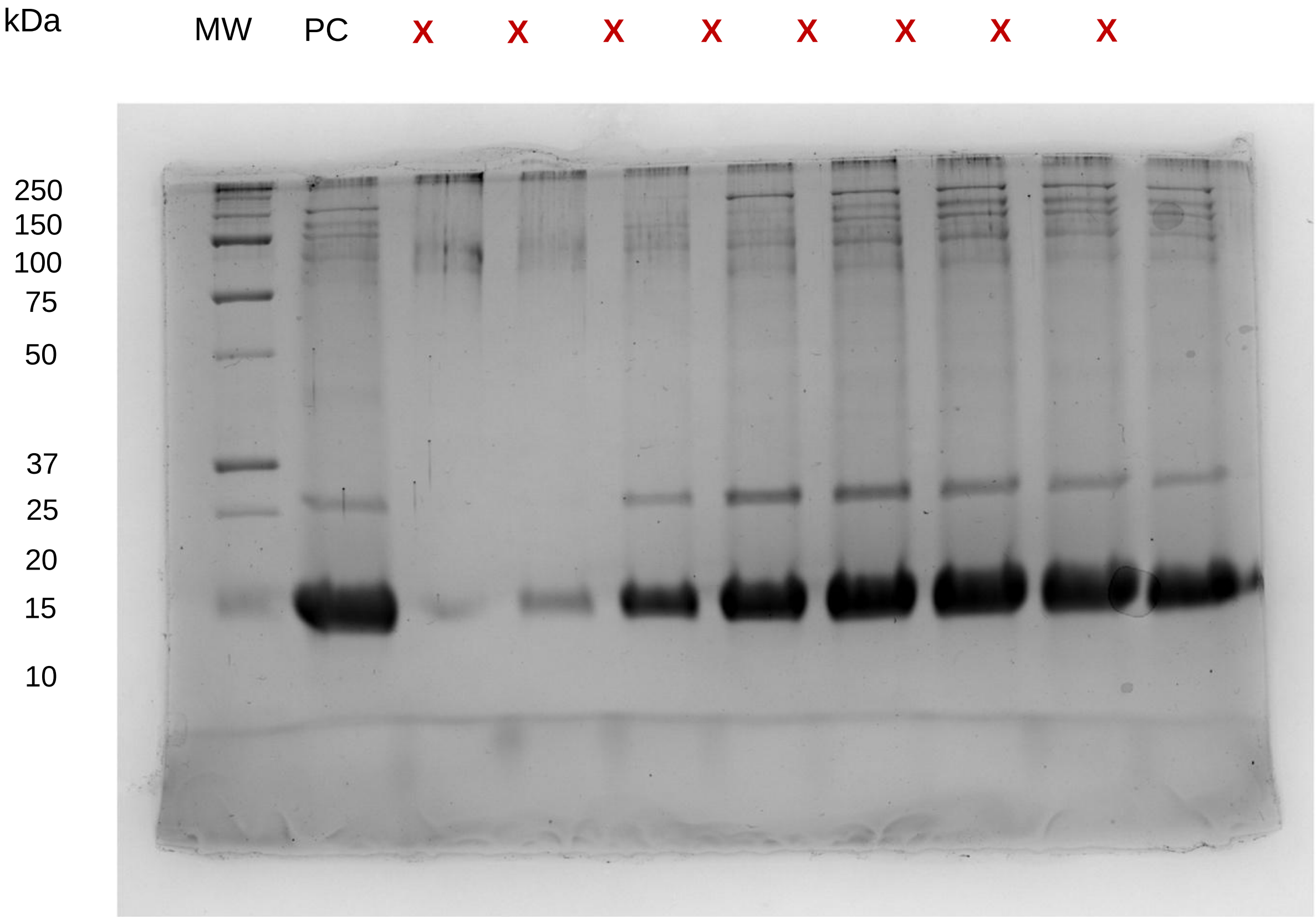

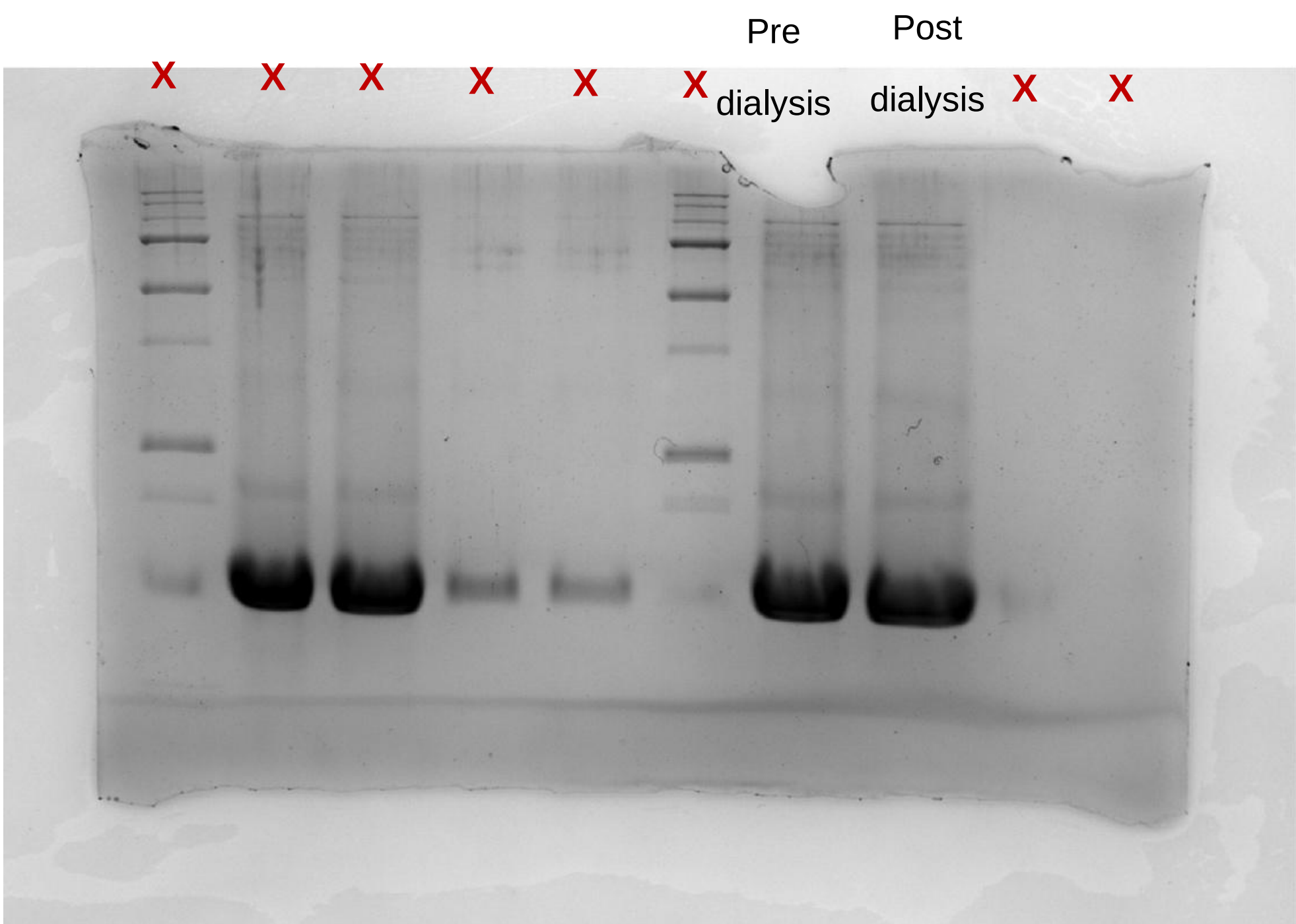

J. Raw image for Figure 3A. Image captured using a Bio-Rad Gel Documentation System

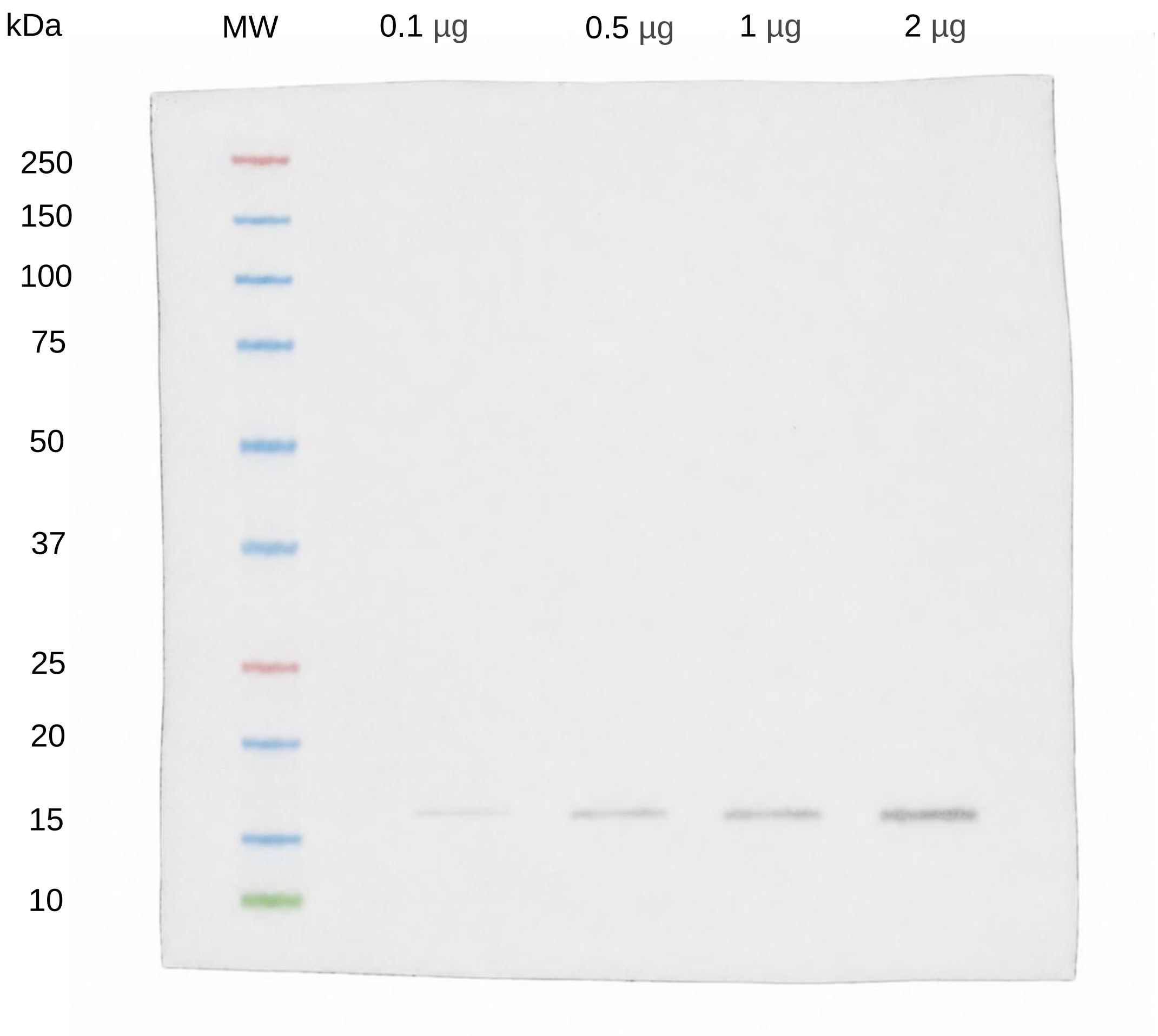

Supplement: Supplementary file 3 [file pntd.0014617.s003.pdf]
